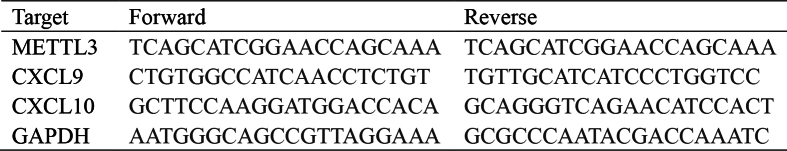# Preventing postsurgical colorectal cancer relapse: A hemostatic hydrogel loaded with METTL3 inhibitor for CAR-NK cell therapy

**DOI:** 10.1016/j.bioactmat.2024.10.015

**Published:** 2024-10-22

**Authors:** Zilin Tan, Liangjie Tian, Yang Luo, Kexin Ai, Xuehua Zhang, Haitao Yuan, Jinfan Zhou, Guangyao Ye, Shuofei Yang, Ming Zhong, Gaohua Li, Yanan Wang

**Affiliations:** a Department of General Surgery, Guangdong Provincial Key Laboratory of Precision Medicine for Gastrointestinal Tumor, Nanfang Hospital, Southern Medical University, Guangzhou, 510515, Guangdong, China; b Division of Orthopaedics and Traumatology, Department of Orthopaedics, Nanfang Hospital Southern Medical University, Guangzhou, Guangdong, 510515, China; c Department of Gastrointestinal Surgery, Renji Hospital, School of Medicine, Shanghai Jiao Tong University, Pujian Road 160, Shanghai, 200127, China; d Department of Hematology, Zhujiang Hospital, Southern Medical University, Guangzhou, China; e Department of Vascular Surgery, Renji Hospital, School of Medicine, Shanghai Jiao Tong University, Pujian Road 160, Shanghai, 200127, China

**Keywords:** Colorectal cancer, CAR-NK cells adoptive therapy, Injectable hydrogel, N6-methyladenosine modification, METTL3

## Abstract

Colorectal cancer (CRC) recurrence post-surgery remains a major challenge. While Chimeric Antigen Receptor (CAR)-engineered natural killer (NK) cells hold immense therapeutic potential, their intratumoral infiltration ability remains limited, hampering efficacy. Building upon prior research suggesting that chemokines like C-X-C motif chemokine ligand 9 (CXCL9) and C-X-C motif chemokine ligand 10 (CXCL10) recruit CAR-NK cells, we hypothesized that tumor cell m6A methylation, regulated by Methyltransferase-like 3 (METTL3), influences chemokine secretion. This study aims to elucidate the underlying mechanisms and improve METTL3 inhibition efficiency. We designed an adhesive hemostasis hydrogel loaded with STM2457, a METTL3 inhibitor, aimed at sustained release in the acidic tumor microenvironment. In vitro, the hydrogel promoted CAR-NK cell recruitment and tumor killing via sustained METTL3 inhibition. The hydrogel's Schiff base bonds further enabled intestinal adhesion and hemostasis in an incomplete tumor resection model of CRC. Combining the hydrogel with CAR-NK cell therapy significantly reduced CRC recurrence in vivo. Overall, our study reveals the crucial role of METTL3 in CRC recurrence and proposes a promising, multimodal strategy using STM2457-loaded hydrogel and CAR-NK cells for enhanced therapeutic efficacy.

## Highlights

• An in situ injection hydrogel for colorectal cancer after resection was developed.
• The gel has excellent adhesive and hemostatic properties for clinical application.
• The gel can regulate m6A methylation and accumulate CAR-NK cells to kill cancer.

## Graphical abstract

This schematic illustrates the process of preparing GOSM-gel and the combination therapy strategy involving tumor excision, GOSM-gel administration, and CAR-NK cells injection for CRC. GOSM-gel, injected at the tumor resection site margin, forms an adhesive gel at the targeted location. The in-situ production of the hydrogel is triggered by the upregulation of CXCL9 and CXCL10 through the downregulation of METTL3, enhancing the therapeutic efficacy of CAR-NK cells infusion.

**Figure:**
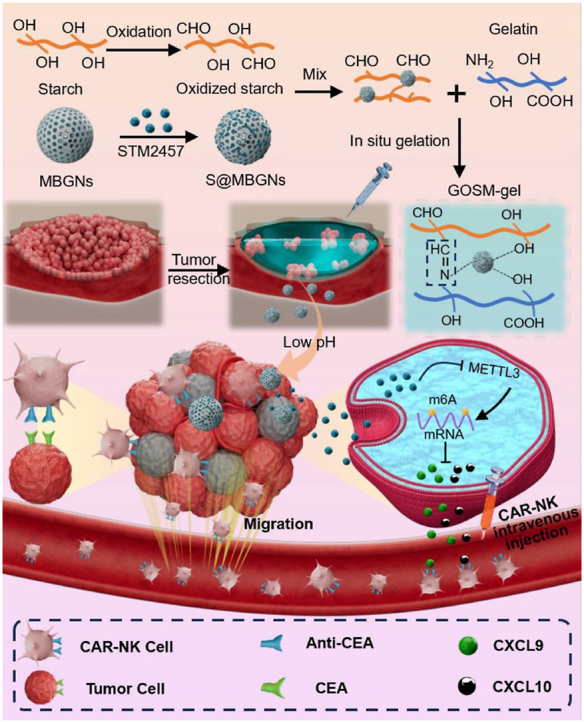

## 1 Introduction

Despite being the second leading cause of cancer-related mortality globally, therapeutic options for colorectal cancer (CRC) remain limited [1]. While surgical resection with adjuvant chemotherapy and radiotherapy represents the standard of care [2], over 30 % of patients with resectable CRC experience relapse, highlighting the critical need for improved prevention strategies [[3], [4], [5]]. This persistent challenge underscores the urgency for novel approaches to effectively prevent postoperative recurrence and metastasis in this patient population [6].

Natural killer cells (NK cells), as part of the innate immune system, play a crucial role in restraining tumor growth and preventing metastatic spread. Notably, their ability to target tumor cells independently of major histocompatibility complex (MHC), especially with the advent of chimeric antigen receptor-engineered NK cells (CAR-NK cells), has spurred significant research in human cancer immunotherapy [7]. Current clinical trials are actively exploring the effectiveness of adoptive CAR-NK cell therapy in cancer treatment, particularly in solid tumors like CRC [8]. Despite the promise of CAR-NK cells, their widespread application faces challenges, including limited proliferative capacity and unstable CAR expression in most cell sources [9]. Induced pluripotent stem cells (iPSCs) have recently emerged as a promising solution to address these limitations [10,11]. Leveraging iPSCs as a source for CAR-NK cells holds potential advantages in overcoming proliferative constraints and ensuring stable CAR expression. Building on these insights, we hypothesize that the infusion of iPSCs-derived CAR-NK cells, exhibiting target specificity against CRC, could represent a promising strategy for CRC treatment. While adoptive CAR-NK cell infusion has demonstrated efficacy in hematological malignancies[[12], [13], [14]], its therapeutic impact in solid tumors, including CRC, has been hindered by challenges such as limited intratumoral accumulation of CAR-NK cells [15]. Therefore, developing novel strategies to enhance the trafficking of CAR-NK cells to tumor sites is pivotal for future development in this field.

According to reports, CXC chemokine ligand 9 (CXCL9) and CXC chemokine ligand 10 (CXCL10) are chemokines that facilitate the migration of NK cells [16]. Notably, it has been reported that the expression of these chemokines, specifically CXCL9 and CXCL10, is upregulated in the tumor microenvironment of METTL3-deficient tumors [17]. The regulation of N6-methyladenosine (m6A) has gained significant momentum in cancer research in recent years [18,19]. This process involves the catalytic activity of writer proteins (METTL3 and METTL14), which modify RNA through m6A modification. In contrast, the eraser proteins, namely the enzymes FTO and ALKBH5, are accountable for the removal of the methyl group. Consequently, the infusion of CAR-NK cells in combination with STM2457 (an inhibitor of METTL3) could present a more optimized treatment strategy for CRC. However, there is currently no information on the long-term inhibition of m6A methylation in tumor cells as a therapeutic approach to enhance the efficacy of CAR-NK treatment in CRC.

In this study, we addressed the aforementioned challenge by developing an adhesive hemostatic hydrogel loaded with a drug for long-term sustained release. Initially, we established a correlation between the high expression of the m6A methyltransferase METTL3 in colon cancer patient samples and poor prognosis, coupled with low expression of chemokines. The field of tumor treatment has witnessed a surge in advancements due to the rising interest in nanocarriers, especially those encapsulated within hydrogels [[20], [21], [22]], driven by their demonstrated efficacy in therapeutic outcomes, as evidenced by numerous studies [23,24]. Building upon this knowledge, we engineered an adhesive hemostatic hydrogel functioning as a dual pH-responsive biomaterial for sustained delivery of STM2457. This design harnesses the acidic TME pH for triggered release and enhanced therapeutic efficacy. This innovation aims to enhance adoptive CAR-NK cell treatment, thereby preventing colorectal cancer recurrence following surgical excision. Drawing from previous research [25,26], our study incorporated biocompatible mesoporous bioactive glass nanoparticles (MBGNs) as drug nanocarriers, capable of responding to the acidic tumor microenvironment. Through the combination of Schiff base interactions between gelatine and oxidized starch (OS), the resulting hydrogel exhibited excellent tissue adhesion, hemostasis, and biocompatibility. STM2457 was initially loaded into MBGNs to create STM2457@MBGNs, which were subsequently integrated into OS. This composite material was then combined with gel to form the gel-OS/STM2457@MBGN hydrogel (GOSM-gel). The GOSM-gel, when injected into the post-surgical site, adheres to living tissue, reducing bleeding volume and time. Furthermore, the GOSM-gel facilitated the gradual release of STM2457 in response to pH changes, creating a conducive environment for CAR-NK cells trafficking into the tumor (Fig. 1). In summary, the GOSM-gel presents a treatment strategy based on m6A methylation regulation, synergistically combined with CAR-NK cells to effectively prevent post-resection CRC recurrence.

**Fig. 1:**
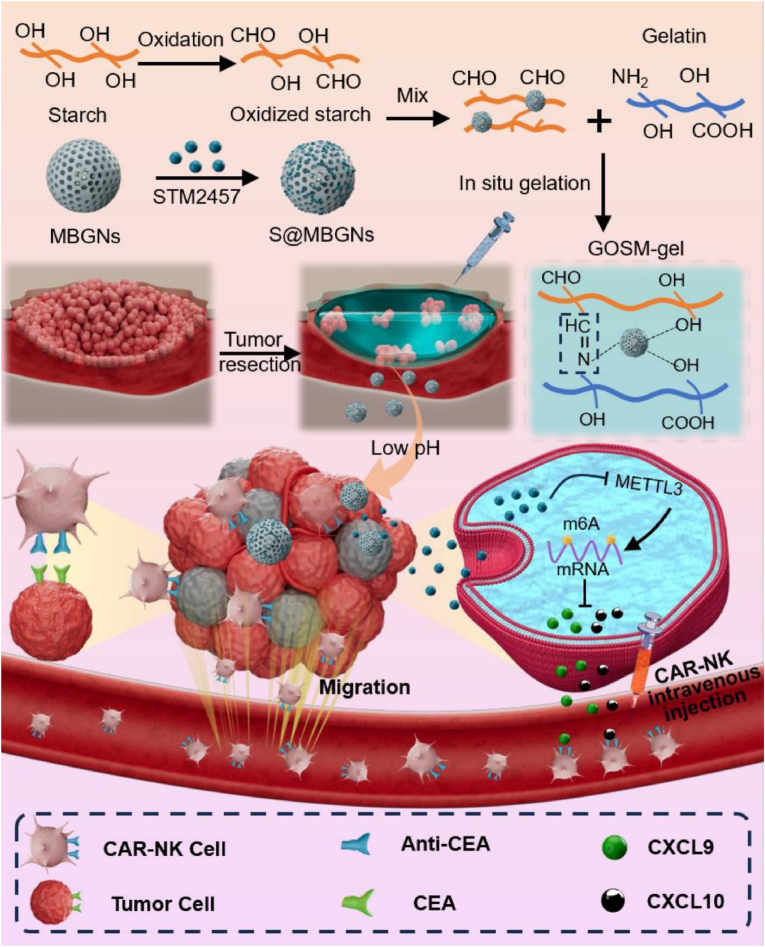
This schematic illustrates the process of preparing GOSM-gel and the combination therapy strategy involving tumor excision, GOSM-gel administration, and CAR-NK cell injection for CRC. GOSM gel, injected at the tumor resection site margin, forms an adhesive gel at the targeted location. The in-situ production of the hydrogel is triggered by the upregulation of CXCL9 and CXCL10 through the downregulation of METTL3, enhancing the therapeutic efficacy of CAR-NK cell infusion.

## 2 Materials and methods

### 2.1 Patients and specimens

This study received approval from the Institutional Review Board of Southern Medical University. CRC patients were recruited by Nanfang Hospital between 2023 and 2024. Before recruitment, all participants provided informed consent.

### 2.2 Materials and reagents

Gelatin (type B, gel strength ∼100 g Bloom), ammonia soluble starch, sodium periodate (AR, ≥99.5 %), ammonia solution (AR, 25–28 %), Ethyl acetate (EA, AR, ≥99.5 %), hexadecyl trimethyl ammonium bromide (CTAB, AR, ≥99 %), 4,6-diamidino-2-phenylindole (DAPI), fluorescein isothiocyanate (FITC), rhodamine b, Cyanine 5.5 (Cy5.5), Cyanine 7 (Cy7), and absolute ethanol (AR, ≥99.7 %) were procured from Aladdin (Shanghai, China). Calcium nitrate (CN, AR, 99 %) was obtained from Tianjin Fuchen Chemical Reagent Factory. STM2457 was purchased from Med Chem Express, STM2457 was dissolved by dimethyl sulfoxide (DMSO) into a 20 mM stock solution. CXCL9 and CXCL10 ELISA kits were acquired from Abcam (Cambridge, UK).

### 2.3 Cell lines and animals

As previously described, induced pluripotent stem cells (iPSCs) generated from umbilical cord blood CD34^+^ cells were maintained on mouse embryonic fibroblasts (MEF) [27]. CAR-iPSCs were generated through lentiviral transduction, followed by FACS to select GFP-positive cells. Cells were incubated in a dark humidity 37 °C incubator with 5 % CO_2_. The process of generating NK cells from iPSCs and CAR-iPSCs has been documented in prior studies [28,29]. In summary, this process consists of two stages, stage 1 is hematopoietic differentiation, and stage 2 is differentiation into NK cells. For stage 1, a density of 3000 TrypLE-adapted iPSCs was initiated in 96-well round-bottom plates utilizing APEL medium [29] supplemented with 40 ng/ml human SCF, 20 ng/ml human VEGF, and 20 ng/ml recombinant human BMP-4. Stage 2 begin on day 11 of the hematopoietic differentiation phase, embryoid bodies were transferred to uncoated 24-well plates for NK cell culture conditions. The cells underwent further differentiation into NK cells as detailed in previous reports [30,31], utilizing a cytokine cocktail consisting of 5 ng/mL IL-3 (for the initial week), 10 ng/mL IL-15, 20 ng/mL IL-7, 20 ng/mL SCF, and 10 ng/mL flt3 ligand over a period of 28–32 days. Half-media replacement was conducted on a weekly basis. The fetal human colon (FHC) cell lines, SW837 cell lines and Ls174T cell lines were obtained from the American Type Culture Collection (ATCC). Male SD rats (200–300 g) and female NOD-SCID mice (6 weeks, 18–22 g) were procured from the Laboratory Animal Center of Southern Medical University and Hua Fukang Biological Technology Co. Ltd. (Beijing, China), respectively.

### 2.4 HE (hematoxylin-eosin) and immunohistochemistry (IHC) staining

Paraffin-embedded blocks of samples were prepared following standard protocols. Deparaffinized and rehydrated tissue sections were stained with conventional HE staining for histological examination under an optical microscope. Subsequently, paraffin-embedded tissues were subjected to immunohistochemistry using antibodies against primary antibodies (Table S1), and the results were graded to determine the expression levels of proteins.

### 2.5 Cytotoxicity assay

Utilizing the LDH assay kit (Beyotime, China), the cytotoxicity of CAR-iPSCs-NK cells was assessed. In brief, Ls174T cells or SW837 cells were seeded at a density of 1 × 10^4^ cells per well in a 96-well plate. CAR-iPSCs-NK cells or iPSCs-NK cells were then added at effector-to-target ratios of 1:1, 2.5:1 and 5:1. Subsequently, 20 μL (5 mg/mL) of the maximum release agent of LDH was added, and the mixture was incubated for 4 h at 37 °C. Absorbance at 490/630 nm was measured using a microplate reader (BioTeK, USA). Ultimately, the following formula was used to determine the percentage of cytotoxicity (Equation (1)):

(1) $$Cytotoxicity=\frac{\left(experimental-target\;spontaneous\right)}{\left(maximum-spontaneous\right)}\times 100\%$$

### 2.6 m6A dot blot assay

Following denaturation at 65 °C for 5min, the samples were spotted onto Amersham Hybond-N+ membrane (GE Healthcare) and cross-linked using UV light. The membrane was then cleansed with Phosphate Buffered Saline with Tween 20 (PBST). The primary antibody used was anti-m6A (202003, Synaptic Systems), and Methylene Blue (MB) served as a loading control.

### 2.7 Assay of carcinoembryonic antigen (CEA) expression (flow cytometry and immunofluorescence (IF))

FHC, SW837 or Ls174T cells (1 × 10^6^ cells/sample) were fixed and incubated with FITC-conjugated CEA (1:20 dilution, BioLegend). Cell surface marker quantification was performed using a Guava easyCyte flow cytometer. For immunofluorescence, FHC, SW837 or Ls174T cells (1 × 10^5^ cells) were fixed in 4 % paraformaldehyde, blocked with 2 % BSA, and incubated with CEA antibodies. Fluorescein-conjugated secondary antibodies were used, and images were captured using a Leica TCS SP8 X confocal microscope.

### 2.8 RNA isolation and qPCR

Samples treated with different groups were lysed using Trizol reagent (Takara, Japan). Total RNA extraction and purification were performed with a FastPure® Cell/Tissue Total RNA Isolation Kit (Vazyme, China). RNA concentration was quantified using a NanoDrop Microvolume Spectrophotometer (Thermo Fisher, USA). cDNA synthesis and qPCR were carried out with the QuantStudio 5 Real-Time PCR System (Japan). Experimental primer details are provided in Table S2.

### 2.9 Western blot analysis

Proteins separated by electrophoresis on a 4–12 % SDS-PAGE gel were transferred to polyvinylidene difluoride membranes (Millipore, USA). After incubation in a 5 % skim milk solution, membranes were probed with primary antibodies overnight at 4 °C. Following incubation with secondary antibodies, the samples underwent washing, and detection was carried out using GelView 6000 Pro (China) and SuperSignal West Pico PLUS (Thermo Fisher, USA). Image analysis was performed using ImageJ software.

### 2.10 Analysis of bioinformatics using publicly available databases

The Cancer Genome Atlas (TCGA) and Gene Expression Omnibus (GEO) databases were used for bioinformatics analysis. TCGA database analysis explored METTL3 expression in human CRC tissues compared to normal adjacent tissues. Kaplan-Meier plots from the GEO database assessed the relationship between METTL3 expression and overall survival. Multivariate analyses established correlations between METTL3 expression, baseline characteristics, and overall survival in CRC individuals.

### 2.11 Cell proliferation assay

Cells seeded in 96-well plates (5000 cells per well) were assessed for viability at different intervals using the CCK-8 Kit (Dojindo Molecular Technologies). Absorbance at 450 nm was measured with a BioTek Epoch microplate reader.

### 2.12 Synthesis of OS

A 10.56 % (w/v) solution of sodium periodate was added dropwise to a 50 mL starch solution. The mixture was kept in the dark at 37 °C for 3 h. Subsequently, 5 mL of ethylene alcohol was employed to induce the precipitation of the oxidized products. The starch oxidation was freeze-dried in a freeze drier (FD-10, China) for 7 days after being frozen at 20 °C for 1 day and starch oxidation was evaluated using Fourier transform infrared (FTIR) spectroscopy.

### 2.13 Synthesis of MBGNs

The synthesis of MBGNs was conducted as described in a previous study [32]. Initially, a solution was created by completely dissolving 2.80 g of hexadecyl trimethyl ammonium bromide in 132 mL of deionized water with continuous stirring at 35 °C. Following that, 40 mL ethyl acetate was added to a solution containing hexadecyl trimethyl ammonium bromide. Following 30 min of stirring, 28 mL of ammonia solution (1 mol/L) was introduced into the solution, followed by 15 min of stirring. Following this, 14.40 mL of tetraethyl orthosilicate was added to the mixture, which was then agitated for 30 min before adding 6.52 g of calcium nitrate. Following this procedure, the solution progressively adopted a translucent white coloration; after 4 h of agitation, colloids were formed. Following purification at 8000 rad/s by centrifugation, three times with ethanol and three times with water, the colloidal particles were rinsed. Following an additional 24 h of drying at 60 °C, the particles were pulverized into fine granules using a mortar. Subsequently, MBGNs were obtained by subjecting the powders to a heating rate of 2 °C/min at 700 °C for 3 h, which effectively eliminated the organic compounds and nitrates that had become entangled in the powders. The surface morphology of mesoporous bioactive glass nanoparticles was examined using a transmission electron microscope (TEM).

### 2.14 Capacity for loading drugs into MBGNs

To acquire STM2457-loading MBGNs, FITC-tagged STM2457 was first dissolved in phosphate-buffered saline (PBS) at a pH of 7.4. Then, the MBGNs were introduced into the solution. Subsequently, the combination underwent vigorous stirring for a duration of 24 h at room temperature. Next, the liquid portion above the sediment was eliminated by subjecting it to centrifugal force for a duration of 10 min at a speed of 9000 revolutions per minute. Subsequently, the precipitate was carefully washed twice using 10 mL of deionized water each time. This process aimed to effectively cleanse the unloaded STM2457. Prior to and following drug loading procedures, the concentrations of STM2457 in solutions were determined by employing a UV–vis spectrometer (Lambda 35, PerkinElmer) set at a wavelength of 460 nm. The encapsulation efficiency (EE%) and loading capacity (LC) of MBGNs were quantified using established gravimetric methods. EE% was determined as the percentage of the initially added drug (STM2457) successfully encapsulated within the MBGNs, calculated as [(M₀ - M₁)/M₀] × 100 %, where M₀ is the total mass of STM2457 added, and M₁ is the mass of free, unencapsulated STM2457 remaining in the supernatant after loading. LC was then calculated as the mass of encapsulated drug per unit mass of MBGNs, using the formula [(M₀ - M₁)/M_s_], where M_s_ represents the mass of the MBGN carrier.

### 2.15 The synthesis of GOSM-gel

To synthesize the GOSM-gel, an OS solution (10 % w/v) was formed by initially dispersing the OS in an aqueous solution. Then, the OS solution was supplemented with STM2457 and STM2457-loaded MBGNs (5 % w/v). After subjecting the resulting mixture to ultrasonic dispersion at 4 °C for 10 min, the OS solution containing MBGNs was generated. Additionally, at 60 °C, gelatin was dissolved in an aqueous solution to obtain gelatin solution (30 % w/v). This process produced a gelatin solution that possessed a 30 % weight-to-volume ratio. Following that, the gelatin solution and the MBGN-containing OS solution were combined. The resulting mixture was subsequently put into a mold at 37 °C to generate GOSM-gel. The surface morphology of the GOSM-gel was examined under a scanning electron microscopy (SEM).

### 2.16 Rheological test

Experiments on rheology were performed in adherence to previously published studies [33]. The hydrogel's injectability was evaluated by measuring its linear viscosity (η) using the frequency sweep mode. The strain sweep measurements were performed across a scope extending from a strain of 0.1 %–1000 %.

### 2.17 Injectability studies

To assess the injectability of the composite gel, solutions containing OS + STM2457-loaded MBGNs and unfilled gel were separately loaded into syringes. OS + STM2457-MBGNs were labeled with methylene blue (MB) for tracking, while the gel was labeled with rhodamine B. The OS + STM2457-MBGNs solution was then injected into an aqueous solution using a three-way tube to evaluate the compatibility and flow behavior of the combined mixture.

### 2.18 Self-healing performance

The test was conducted by cutting two gels that had been dyed blue and red, respectively. After 20 min of healing, two gels were reconnected after cutting. Tensile stress was applied to the reunited sample to determine its ability to mend itself.

### 2.19 Phagocytosis assay

In order to examine the process of phagocytosis, the MBGNs dyed with Rhodamine b and STM2457 dyed with FITC-labeled. Ls174T cells were cultured on MBGNs that were loaded with STM2457. The cells were then fixed with 4 % paraformaldehyde and stained with a nuclear stain (DAPI).

### 2.20 The degradation and biocompatibility of GOSM-gel

Blood compatibility testing was done to assess the biocompatibility of the GOSM-gel as previously described [34]. In summary, a total of 500 μL of pre-treated blood with sodium citrate anticoagulant was mixed with 100 μL of GOSM-gel, PBS, or 1 % Triton X-100. Following this, the blood samples were subjected to agitation in a shaker at a temperature of 37 °C for a duration of 3 h. Subsequently, the blood sample underwent centrifugation at a speed of 4000 rpm for 10 min. Following centrifugation, a volume of 200 μL of the resulting supernatant was collected, to which 5 mL of deionized water was subsequently introduced. In this study, the absorbance at a wavelength of 540 nm was measured using UV–vis spectroscopy. Subsequently, the hemolysis ratio was determined using the obtained absorbance data. Furthermore, the hydrogel's biocompatibility was assessed by using tests for cell viability and live/dead staining. A preliminary volume of 100 μL of hydrogel was introduced into the lower wells of 96-well plates for this experiment. Following this, the plates underwent an incubation period of 4 h, after which they were rinsed three times with PBS. Subsequently, FHC were seeded onto the hydrogel surfaces at a volume of 100 μL per well, with 6000 cells per well. After that, the cells were cultivated for 24 h at 37 °C with 5 % CO_2_. After the time of incubation, the assessment of cell viability was conducted using a CCK-8 kit. Cell viability was assessed via Live/Dead staining. Briefly, 6-well culture plates were seeded with 1 × 10^5^ FHC cells/well. Following a 24-h co-incubation with samples from each experimental group, cells were treated with a Live/Dead staining solution prepared by mixing 1 mL PBS, 3 μL Calcein AM, and 5 μL propidium iodide (PI) from Invitrogen. Incubation with the staining solution proceeded at 37 °C for 30 min. The samples were subsequently examined through the utilization of an inverted fluorescence microscope (Olympus, BX63, Japan). To assess the biodegradation process, the hydrogel was initially submerged in a 5 mL solution of PBS with pH 7.4 and pH 6.5, maintained at a temperature of 37 °C. Then, the mixture was swirled at a constant 90 revolutions per minute. The hydrogel that was left over was then rinsed with a PBS solution, and their weights were recorded while they were still wet. The degrading ability of the hydrogel in vivo was assessed using the previously established study [35,36]. In this study, gelatin was initially labeled with Cy7, after which the hydrogel was created utilizing the gelatin that had been labeled with Cy7. Subsequently, the hydrogel was administered to animals by subcutaneous injection, and the subsequent degradation of the hydrogel was seen by monitoring the Cy7 signal using an in vivo fluorescent imaging system. Furthermore, an additional assessment was conducted to examine the degradation behavior of hydrogel 1, 7, 14, and 21 days following their subcutaneous injection into mice. To evaluate the in vivo compatibility of GOSM-gel, we injected GOSM-gel subcutaneously into mice while injecting PBS subcutaneously into a control group of mice. Blood samples were obtained at day 14 following therapy administration. The purpose of collecting these samples was to investigate the biomarkers associated with liver function, kidney function, and the proportion of blood cells.

### 2.21 GOSM-gel drug release

To study the in vitro drug release characteristics, a hydrogel was created using FITC-labeled STM2457. The procedure is similar with prior studies [37], STM2457 and FITC were dissolved in DMSO at a defined ratio and agitated overnight under dark conditions. Subsequently, the surplus FITC was eliminated via dialysis. Finally, the FITC-labeled STM2457 was acquired post freeze-drying in the absence of light. The hydrogel was immersed in a PBS solution with pH 7.4 or 6.5 and gently agitated at 37 °C. The liquid portion of the mixture was collected and replaced at designated intervals. UV–Vis–NIR absorbance spectrum was employed to quantitatively assess the drug released in the supernatant. For the in vivo assessment of drug release, the previously established protocol was followed [35,36]. Cy5.5-labeled STM2457 was used to fabricate a gel known as GOSM-gel, and STM2457 was dispersed within the precursor solution of GO-gel, serving as the control group. These gels were injected into the tumor resection site of CRC. A fluorescent imaging device then detected the presence of Cy5.5-tagged STM2457 within the remaining gel for monitoring purposes. To evaluate the distribution of STM2457 in tumor tissue, tumor samples were collected from mice at four different time points (day 1, 7, 14, and 21) after applying GOSM-gel or receiving an intravenous injection of STM2457. Frozen slices were obtained from these samples, and the signal emitted by the FITC-labeled sample was examined using a confocal laser scanning microscope. Additionally, referencing previous report [38], serum concentrations of STM2457 were quantified using HPLC on blood samples collected from mice at specified time intervals. Centrifugation was employed to separate plasma components, followed by the combination of 20 μL plasma with a 120 μL acetonitrile precipitant solution containing an internal standard. The resulting supernatant was then diluted with water at a ratio of 1:1 (v/v), and 2.5 μL of this solution was analyzed using liquid chromatography-tandem mass spectrometry (LC-MS). Chromatographic separation was achieved on a Hypersil Gold C18 column (30 mm × 2.1 mm, 1.9 μm particle size) with a gradient elution from 5 % to 95 % solvent B over a 30-s period. The mobile phase was composed of water containing 0.1 % formic acid (solvent A) and acetonitrile (solvent B), with a flow rate maintained at 1.0 mL/min. The blood to plasma concentration ratio was ascertained from relevant sample data, and the lower limit of quantification (LLOQ) was established at 10 ng/mL.

### 2.22 Determination of m6A level in Ls174Ts

Ls174Ts cells were seeded overnight in six-well plates at a density of 1 × 10^5^ cells per well. Subsequently, the cells were divided into four distinct groups, and each group was subjected to different treatments: PBS, GOM-gel, STM2457, and GOSM-gel. After a 24-h incubation period, total RNA was extracted from Ls174T cells. The m6A concentration in the RNA was determined using the EpiQuik m6A RNA Methylation Quantification Kit (EpiGentek, Farmingdale, NY, USA), following the manufacturer's guidelines.

### 2.23 Assay for colony formation

500 cells were seeded into each well of a 6-well plate. To assess the colony-forming ability after approximately 7 days, cells were fixed with 4 % paraformaldehyde and stained with a 1 % crystal violet solution.

### 2.24 Quantification of chemokines in Ls174T supernatants and serum using ELISA

The chemokine levels (CXCL9 and CXCL10) were ascertained utilizing Enzyme-Linked Immunosorbent Assay (ELISA) Duoset kits provided by R&D Systems Inc. (Minneapolis, MN, USA) on the collected supernatants and serum. The absorbance wavelength of 450 nm was utilized to quantify the color intensity through the utilization of a BioTek ELx808 plate reader (BioTek Instruments, Winooski, VT, USA).

### 2.25 In vitro chemotaxis assay

To conduct a chemotaxis assay for NK-92 cells migration, A total of 2 × 10^5^ NK-92 cells were placed in the upper chamber of a 24-well transwell plate with 100 μL of serum free-medium. The lower chamber of a 24-well Transwell plate (Corning, USA) with 8 μm pores received 600 μL of conditioned media harvested from the supernatants of distinct experimental groups of Ls174T cells. The cells were cultured for 8 h at 37 °C and 5 % CO_2_ in an incubator. At the designated endpoint, the abundance of NK-92 cells within the lower chamber was assessed.

### 2.26 In vitro adhesion and burst pressure tests

The hydrogel precursor solution was evenly applied to the interstitial space between two sections of porcine skin, each measuring 20 mm in length and 10 mm in width. The hydrogel-crosslinked connection spanning a 1 cm^2^ area between the two pig skin sections was subjected to a lap-shear test using a dynamic mechanical analyzer (DMA Q800, USA) at intervals of 20 min, with a testing speed of 1 mm/min. Additionally, a burst test was conducted using a custom-designed apparatus previously employed in our earlier study to assess the hydrogel's effectiveness as a tissue sealant.

### 2.27 In vivo hemostatic effect testing

As previously documented, the hemostatic activity of the hydrogel was evaluated in vivo utilizing two rat bleeding models: a model of rat tail amputation hemorrhage and the rat partial hepatectomy hemorrhage model [34]. The quantification of blood loss within a 2-min timeframe following liver resection was performed for each group, and the duration of hemostasis was recorded until cessation of bleeding.

### 2.28 The efficacy of GOSM-gel on in situ colorectal cancer mice model

To develop a C57BL/6 mouse model for orthotopic colorectal cancer using MC38-luc cells, 2 × 10^6^ cells were orthotopically implanted into the cecal wall of 6–8 weeks old female mice. Postoperative care was administered, and the abdomen was closed with sutures. On day 7, various treatments were administered into the cecal tumor: PBS as a control, GOM-gel, STM2457, and GOSM-gel. The dose of STM2457 in each group was equal (50 mg/kg). This dosage was chosen based on previous studies that have established the efficacy and tolerability of this dose in mouse models [39]. Tumor progression was monitored using an in vivo bioluminescence imaging system. On day 20, tumors were harvested and subjected to flow cytometry analysis, all flow cytometry analysis gating strategy used of flow cytometry results are shown in Fig. S10.

### 2.29 The combined adjuvant therapy's preventive effectiveness in tumor recurrence

70 % of the Ls174T tumor in female NOD/SCID mice was removed once its volume reached 200–300 mm^3^. The mice were then randomly assigned to 5 groups: group 1 received PBS treatment; group 2 received adoptive CAR–NK cell injection; group 3 received GOM–gel and adoptive CAR-NK cell injection; group 4 received an intravenous (IV) injection of STM2457 and adoptive NK cell injection; and group 5 received GOSM–gel and adoptive CAR–NK cell injection. The dose of STM2457 in each group was equal (50 mg/kg). On days 8 and 15, mice were given CAR-NK cells (1 × 10^6^). The in vivo bioluminescence imaging system monitored the recurrence of the tumor, and the luminosity intensity was employed to assess the growth of the tumor. The mice's survival period was measured for 40 days, or until the tumor killed them. Every other day, body weights were simultaneously assessed to assess systemic toxicity.

### 2.30 Statistical analysis

The study presented quantitative data as the mean ± standard deviation (SD) derived from a minimum of three independent experiments. Statistical analyses involved the comparison of data using the student's t-test (two-tailed) and one-way analysis of variance (ANOVA). The overall survival rate of tumor patients was evaluated using the Kaplan-Meier method and compared using the Log-rank test. Statistical significance was determined with ∗*p* < 0.05, ∗∗*p* < 0.01 and ∗∗∗*p* < 0.001, indicating increasing levels of significance. All statistical analyses were conducted using GraphPad Prism 8.0 (GraphPad Software, USA).

## 3 Results and discussion

### 3.1 Characterization and killing ability against tumor of CAR-iPSCs-NK cells and high METTL3 expression is associated with an unfavorable prognosis for patients with CRC

Immunotherapy is undergoing a significant transformation with the adoption of CAR-NK cells, marking a revolutionary advancement in cancer treatment. CAR-NK cells, in comparison to NK cells, exhibit enhanced anticancer properties and the ability to specifically target cancers [27,40]. CEA has emerged as a promising target for cancer immunotherapy due to its widespread expression on the surface of various solid tumor cells, including CRC cells, while being minimally or not expressed on normal cells [41,42]. In our study, we confirmed the positive expression of CEA in colorectal cancer tissues through immunohistochemistry (Fig. S1), followed by validation of its expression in the colorectal cancer cell line (Ls174T) cells using flow cytometry and IF (Figs. S2 and S3). In addition, we selected CEA negative colorectal cancer cell line SW837 as the reverse validation based on previous study [43], and also detected the CEA expression level of SW837 cells through flow cytometry and IF experiments. The results showed that CEA was almost not expressed in FHC and SW837, while highly expressed in Ls174T cells (Figs. S2 and S3). These findings indicated that CEA could serve as a prospective CAR target for CRC. We engineered CAR-iPSCs-NK cells, transforming iPSCs NK cells with a CAR designed to specifically target CEA (Fig. S4). Post-transfection, approximately 81.8 % of anti-CEA iPSCs-CAR-NK cells expressed anti-CEA recombinant NK-cell receptor (Fig. 2a and Fig. S5), exhibiting enhanced cytotoxicity against Ls174T cells compared to iPSCs-NK cells (Fig. 2b). Meanwhile, we validated the cytotoxicity of iPSC NK cells and CAR-IPSC-NK cells against CEA negative SW837 cells, and the results showed no statistically significant difference in their cytotoxicity against SW837 cells (Fig. 2c). These results indicate that the more effective capability of CAR-NK cells in killing CRC are mediated by targeting CEA. This success demonstrated that anti-CEA iPSCs-CAR-NK cells showed more effective lethal activity against CRC cells. To comprehend the role of m6A alteration in CRC, we initially analyzed the levels of m6A RNA in 10 CRC tissues and their corresponding normal colorectal mucosa. Our results revealed significantly elevated m6A RNA levels in CRC tissues, validated through the dot blot method and colorimetric analysis using the m6A RNA methylation quantification kit (Fig. 2d and e). It is well-established that METTL3, METTL14, and WTAP are primary m6A writers, while ALKBH5 and FTO are primary erasers. Reports have consistently identified METTL3 as the key player in m6A modification in CRC[[44], [45], [46], [47]]. Consequently, we compared METTL3 expression levels in 10 pairs of colorectal cancer and normal tissue samples, revealing significant upregulation of METTL3 in CRC through immunohistochemical staining (Fig. 2f and g). This upregulation was further confirmed by qPCR and Western blot (WB), showing a substantial increase in METTL3 expression in human colorectal cancer tissues compared to normal colorectal mucosa (Fig. 2h–j). Furthermore, according to previous research report, the expression levels of chemokines CXCL9 and CXCL10 were negatively correlated with METTL3 [17]. Analysis of TCGA data validated the significant upregulation of METTL3 mRNA in CRC (Fig. 2k). Additionally, data from the GEO database indicated that CRC patients with elevated levels of METTL3 mRNA experienced significantly poorer overall survival rates (n = 52, p < 0.001, Fig. 2l). Multivariate Cox regression analysis identified METTL3 as an independent prognostic indicator for patients with CRC (HR = 1.871, 95 % CI 1.309–2.675, Fig. 2m). These findings underscored the potential of METTL3 as a treatment target for CRC.

**Fig. 2:**
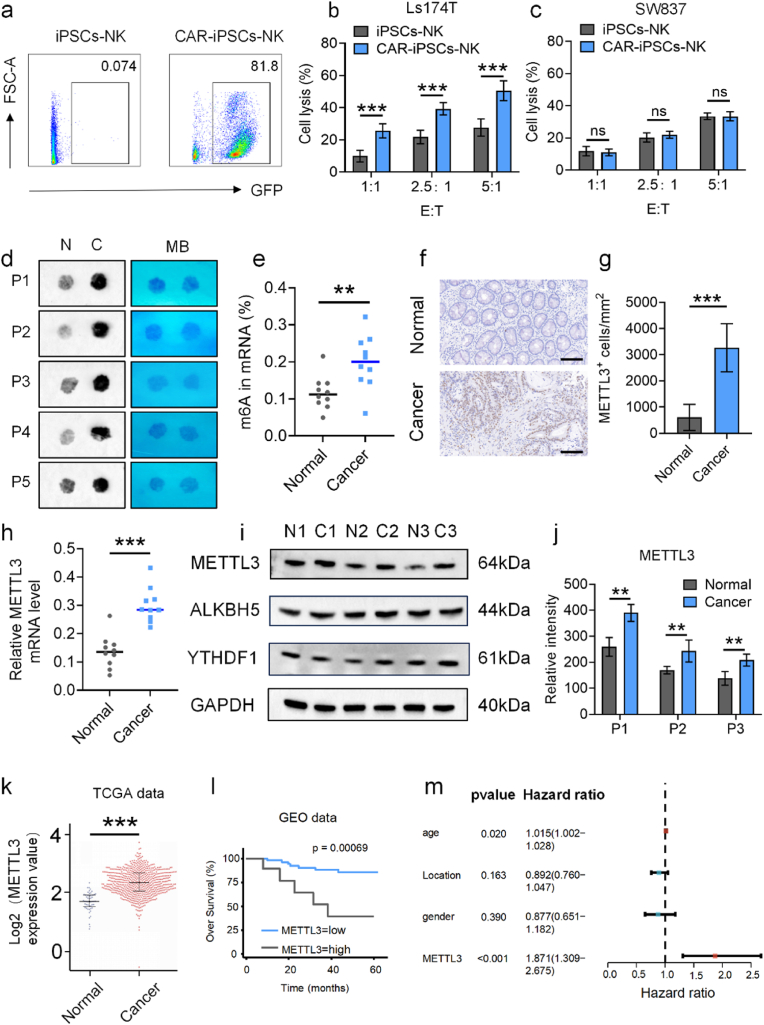
The features of CAR-NK and elevated METTL3 expression is associated with a worse outcome in CRC patients. (a) GFP expression in CAR-iPSCs-NK cells. (b) The relative quantification of GFP^+^ cells ratio in CAR-iPSCs-cells. (c) LDH assay was used to assess the cytolytic activity of anti-CEA CAR-iPSCs-NK cells against Ls174T cells. (d) Dot blot analyses were performed on mRNAs extracted from CRC tissues (C) and normal intestinal mucosa (N) of 5 patients (P) using an anti-m6A antibody, with MB staining serving as the loading control. (e) The methylation quantification reagent for m6A RNA assessed the m6A RNA levels in 10 CRC samples and paired normal intestinal mucosa. (f) By IHC staining, the positive expression of METTL3 was identified in CRC tissues but not in the normal intestinal mucosa (Scale bar: 20 μm). (g) Quantiﬁcation of immunohistochemistry of METTL3 (*n* = 3). (h) qPCR was used to assess the levels of METTL3 expression in paired normal colorectal mucosal tissues and CRC (*n* = 10). (i) METTL3 protein levels were measured in CRC tissues and paired normal intestinal mucosa tissues by WB. (j) Quantiﬁcation of WB of METTL3. (k) Using TCGA data, analyzed were the levels of METTL3 expression in 638 cases of CRC and 51 cases of normal colorectal mucosal tissues. (l) Using GEO data, OS Kaplan-Meier survival curves were constructed by utilizing METTL3 expression data. (m) Multivariable analyses were performed in the CRC cohort. All bars correspond to 95 % CIs. Data are represented as mean ± SD, with significance levels indicated as follows: ∗*p* < 0.05, ∗∗*p* < 0.01 and ∗∗∗*p* < 0.001.

### 3.2 The synthesis and analysis of the GOSM-gel

The compound STM2457, a recently developed METTL3 inhibitor known for its high selectivity and oral bioavailability, was previously explored in a scientific investigation focused on leukemia [38]. In the process of creating the Gel-OS/STM2457@ MBGNs hydrogel, we initially prepared oxidized starch and mesoporous bioactive glass nanoparticles following a procedure outlined in a prior study [48]. FTIR analysis of the starch spectra revealed a previously unrecognized infrared band at a wavelength of 1738 cm^−1^, indicating the presence of aldehyde groups associated with starch oxidation (Fig. S6). TEM displayed microsphere configurations of MBGNs with diameters ranging from 100 to 200 nm (Fig. 3a). Malvern Nano Zetasizer analysis further confirmed the diameter distribution of MBGNs within the range of 100–200 nm (Fig. 3b). In prior investigations, MBGNs were determined to consist of a composition including CaO (15 mol%) and SiO_2_ (85 mol%) [32]. In the present study, FTIR analysis identified a prominent peak of absorption at 812 cm^−1^ and a wide peak ranging from 1300 to 1000 cm^−1^ within the MBGNs spectrum, corresponding to the asymmetric vibrations of the Si-O-Si and Si-O- bonds, respectively (Fig. 3c). The structure of the GO-gel, examined by SEM, is depicted in Fig. 3d. Subsequently, STM2457 was introduced onto MBGNs to generate STM2457-loaded MBGNs, denoted as STM2457@MBGNs. The encapsulation efficiency of STM2457 was 22.9 %, with a loading capacity of 60.6 %. GOSM-gel was created by mixing 30 % (w/v) of Gel with 10 % (w/v) of OS solutions containing STM2457@MBGNs (5 % (w/v)) at 37 °C (Fig. 3e). A control group, GO-gel, was established without MBGNs by combining Gel (30 % (w/v)) and OS (10 % (w/v)). Using dynamic time sweep experiments in rheology, the gelation process of GOSM-gel was monitored, with gelation indicated by the crossings of G″ and G′ (Fig. 3f).

**Fig. 3:**
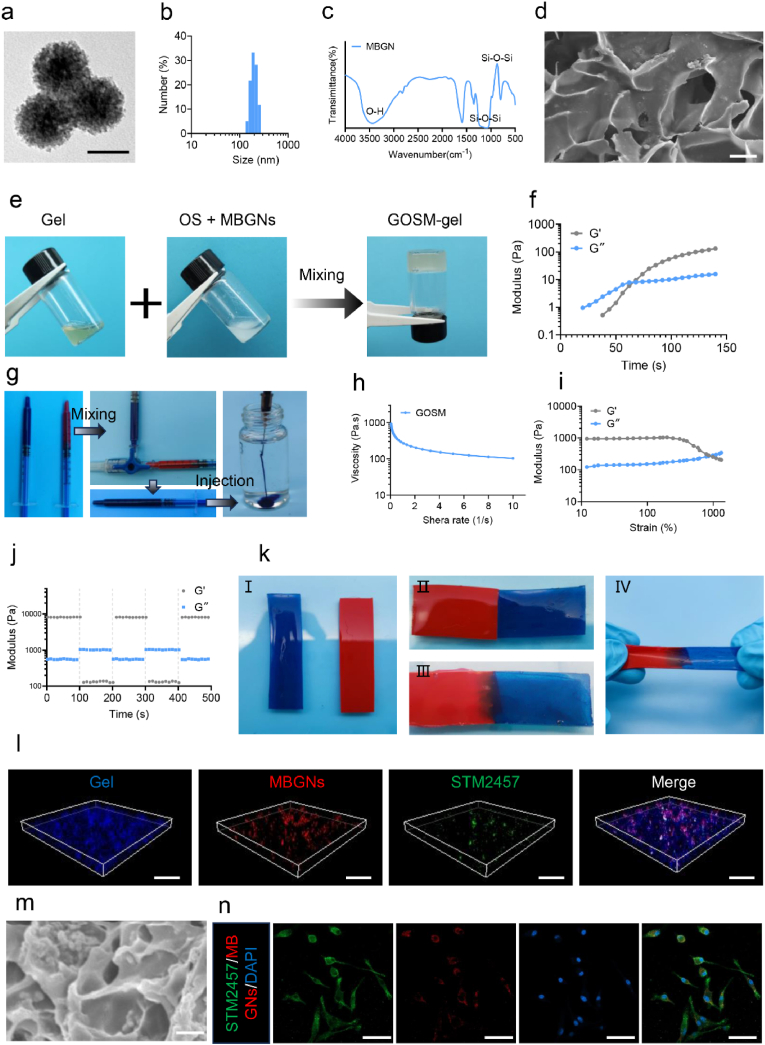
Characterization of GOSM-gel. (a) Images of MBGNs captured by a TEM (Scale bar: 100 nm). (b) The mean hydrodynamic size of MBGNs as established by UK analysis. (c) FTIR spectra of MBGNs. (d) SEM images of GO-gel (scale bar: 2 μm). (e) Photographs illustrating the GOSM-gel in a glass vessel, composed of gelatin (30 % (w/v)) and OS (10 % (w/v)) containing MBGNs (5 % (w/v)). (f) A dynamic time sweep rheological analysis was performed to evaluate the kinetics of gelation for the GOSM-gel. The intersection of G″ and G′ is considered as the gelation time. (g) Injectability of GOSM-gel. (h) GOSM-gel shear-thinning test. (i) The strain amplitude sweep test. (j) Verification of the quick self-healing ability of GOSM-gel by continuous step strain measurements. (k) Images showing the self-healing characteristics of the hydrogel. (l) Representative fluorescent images of a GOSM-gel, where gelatine was stained with DAPI, MBGNs were labeled with rhodamine b, and STM2457 was labeled with FITC (Scale bar: 5 μm). (m) SEM image of the GOSM-gel (Scale bar: 1 μm). (n) IF images demonstrated that the intensity of the green fluorescence emitted by STM2457 was spatially coincident with the red fluorescence emitted by MBGNs in the Ls174T (Scale bar: 50 μm).

Significantly, the GOSM-gel precursor exhibited a high degree of compatibility with water, allowing for seamless injection using a syringe and resulting in the formation of a stable gel (Fig. 3g). This observation suggested that the GOSM-gel possessed favorable injectability characteristics. Subsequently, the viscosity of the GOSM-gel was quantified using a rheometer to conduct a more comprehensive assessment of its injectability. According to the data presented in Fig. 3h, the viscosity of GOSM-gel decreased gradually as the shear rate increased. This indicated that the dynamic hydrogel network of GOSM-gel was susceptible to shear-induced breakdown, potentially enabling the gel to be injected. Hydrogels typically encounter external mechanical forces when used on injured tissues, which might compromise their structural integrity, reduce their longevity, and impact their functional capabilities [33]. The remarkable capacity for self-healing enabled the hydrogel to effectively respond to external mechanical forces, significantly extending its longevity. Therefore, strain amplitude sweep measurements were performed to assess the self-healing capability of the GOSM-gel at a temperature of 37 °C. The minimal variation observed in G″ and G′ levels of GOSM-gel until the strain reached 10 %, as illustrated in Fig. 3i, indicated that GOSM-gel possessed a significant capacity for elastic deformation. However, as the strain continued to increase, there was a significant reduction in the values of the loss modulus (G″) and storage modulus (G′). Ultimately, when the strain reached 100 %, these values were identical. The results suggested that the GOSM-gel exhibited the capability to change state into a solution when the network's critical strain was surpassed. Subsequently, a continuous cyclic strain test was conducted on the GOSM-gel to assess its recoverability, employing strain levels of 5 %, 300 %, and 5 % in a cyclical manner. The experiment was conducted with a consistent angular frequency of 1 rad/s. As depicted in Fig. 3j, when subjected to a strain of 300 % that surpassed the critical strain of 100 %, the GOSM-gel underwent a transition into a sol state, characterized by a G′ value that was lower than the G″ value. Nevertheless, the values of G′ and G″ reverted to their initial values without any diminishment, and the gel-like properties of the GOSM-gel were reinstated when the strain was reduced to 5 %. Moreover, the self-healing capacity of GOSM-gel at 37 °C was evaluated (Fig. 3k). The data reported in the above study confirmed that the GOSM-gel material exhibited rapid gelation when exposed to external mechanical forces, suggesting exceptional self-healing capabilities. To visualize STM2457 loaded on MBGNs, gelatin was labeled with DAPI dyes, MBGNs were labeled with rhodamine b dyes, and STM2457 was labeled with FITC dyes, followed by visualization under confocal reflection microscopy. The 3D IF assay exhibited the homogeneous spatial distribution of STM2457 on the surface of the MBGNs, revealing the successful loading of GOSM-gel (Fig. 3l). As shown in Fig. 3m, GOSM-gel exhibited a characteristic porous morphology with rough pore surfaces, attributed to the presence of attached MBGNs. Besides, the green fluorescence released by STM2457 exhibited a strong correlation with the red signal of MBGNs in the cellular area (Fig. 3n). Taken together, the aforementioned findings suggested that the fabrication of GOSM-gel was accomplished successfully.

### 3.3 Biocompatibility and biodegradation of the GOSM-gel

To evaluate the hemocompatibility of GOSM-gel, hemolysis studies were conducted using PBS as the negative control and Triton X-100 as the positive control. As illustrated in Fig. 4a, the Triton X-100 group's solution displayed a red coloration, indicating blood cell rupture. In contrast, the GOSM-gel group's solution exhibited brightness comparable to the negative control group. In the quantitative analysis presented in Fig. 4b, both the GOSM-gel and PBS groups showed blood loss rates of less than 5 %. This result suggests a high level of hemocompatibility in both groups. Thus, the hemocompatibility of GOSM-gel was confirmed by the obtained results. For cell viability studies and live/dead staining experiments, the FHC cells were cultured in 96-well and 6-well plates coated with GOSM-gel or left uncoated. As shown in Fig. 4c, the disparity in cellular viability between the GOSM-gel group and the control group was not statistically significant. After a 2-day incubation period, both the GOSM-gel and control group displayed a consistent and even dispersion of viable green cells across the entire observed area (Fig. 4d). GOSM-gel exhibited no significant detrimental effect on cellular viability, suggesting its biocompatibility. Given the importance of the biodegradability of implanted biomaterials, a thorough evaluation of the biodegradation characteristics of GOSM-gel was undertaken. The in vivo degradation of GOSM-gel was evaluated by subcutaneously injecting Cy7-labeled GOSM-gel into the dorsal region of rodents and monitoring its fluorescence signal over time. After a 21-day injection period, detectable Cy7 signal remained minimal (Fig. 4e and f), suggesting a gradual decline in the Cy7 signal emanating from the hydrogel. Additionally, monitoring of the implanted hydrogel was performed at designated time intervals. The volume of the hydrogel also exhibited a gradual reduction, reaching a nearly imperceptible level by day 21 (Fig. S7). In vitro degradation studies of GOSM-gel were conducted under distinct conditions, specifically in pH 6.5 and pH 7.4 PBS solutions at a temperature of 37 °C. As depicted in Fig. 4g, both pH 7.4 and pH 6.5 show a steady deterioration of the GOSM-gel. The disintegration rate of GOSM-gel was much slower in pH 7.4 PBS compared to pH 6.5 PBS. Specifically, in pH 7.4 PBS, the weight ratio of the remaining GOSM-gel dropped to 38.9 % after 21 days, whereas it decreased to 5.93 % in pH 6.5 PBS. In summary, the results from this research suggest that GOSM-gel exhibits desirable biodegradability properties. Subsequently, to conduct a more comprehensive assessment of the biocompatibility of GOSM-gel in an in vivo setting, subcutaneous injections of GOSM-gel were administered to mice. The control group in this experiment consisted of mice injected subcutaneously with PBS. For biochemical examination, blood samples were obtained from rodents in both the control group and the GOSM-gel group. According to the findings presented in Fig. 4h, no significant differences were observed between the control group and the GOSM-gel group in the concentrations of aspartate aminotransferase (AST), alanine aminotransferase (ALT), UREA, and creatinine (CREA). This indicates that the administration of GOSM-gel does not adversely affect kidney and liver function. Additionally, the analysis of white blood cell (WBC) count, hemoglobin (HGB), red blood cell (RBC) count, platelet (PLT) count, hematocrit (HCT), and the percentage of granulocyte (gran %) in mouse blood suggests that GOSM-gel did not exhibit hematologic toxicity. Furthermore, pathological exams were conducted to assess potential tissue damage caused by GOSM-gel in various organs of mice, including the liver, kidney, heart, spleen, and lung. The findings, as illustrated in Fig. 4i, suggested that GOSM-gel did not cause major organ toxicity. In summary, the results of this study confirm that GOSM-gel possesses a satisfactory level of biodegradation capability.

**Fig. 4:**
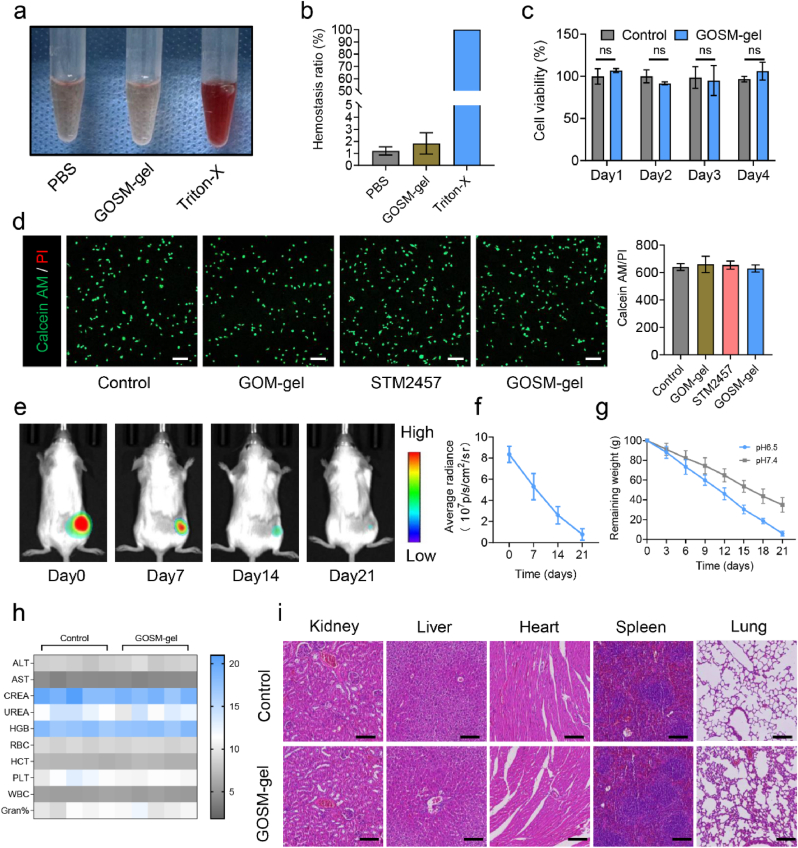
Biocompatibility and biodegradation of GOSM-gel. (a) GOSM-gel hemocompatibility test representative images. (b) GOSM-gel hemocompatibility test quantitative results (*n* = 3). (c) Cytocompatibility test quantitative results (*n* = 3). (d) GOSM-gel staining images of FHC cells after 24 h of contact (scale bar: 200 μm). (e) Fluorescence IVIS imaging for the in vivo degradation of Cy7-labeled GOSM-gel. (f) Cy7-labeled GOSM-gel in vivo retention profile, (*n* = 3). (g) The amount of GOSM-gel that remained after three PBS incubations at pH 6.5 and pH 7.4 (*n* = 3). (h) Blood cell characteristics and indicators of liver and kidney function measured in serum after GOSM-gel was administered subcutaneously to mice (*n* = 5). (i) Histopathological exams of major organs in the mice control and GOSM-gel groups (Scale bar: 25 μm). Data are represented as mean ± SD, with significance levels indicated as follows: ∗*p* < 0.05, ∗∗*p* < 0.01 and ∗∗∗*p* < 0.001.

### 3.4 STM2457 sustained release utilizing the GOSM-gel hydrogel

STM2457 is a highly potent and selective inhibitor of METTL3, exhibiting excellent bioavailability. Emerging evidence supports the ability of STM2457 to reduce m6A levels and hinder the proliferation of various cancer types, including acute myeloid leukemia (AML), SHH-medulloblastoma, and intrahepatic cholangiocarcinoma (10.13039/501100018804ICC) [49,50]. In murine models, IV administration of STM2457 has demonstrated significant antitumor efficacy by modulating RNA m6A levels [38]. Notably, the downregulation of METTL3 expression in CRC has been associated with an upregulation of CXCL9 and CXCL10 expression [17]. This upregulation may facilitate the migration of NK cells, potentially enhancing the therapeutic efficacy of adoptive NK cell therapy. STM2457 holds promise as a means to improve the effectiveness of NK cell infusion in colorectal cancer by promoting NK cell migration into the tumor. However, the development of a new delivery mechanism for STM2457 is crucial due to its very short half-life of less than 6 h in circulation [38]. To evaluate in vivo release characteristics, a GOSM-gel was synthesized incorporating Cy5.5-labeled STM2457. Additionally, Cy5.5-labeled STM2457 was enclosed within a GOS-gel matrix to establish a control group consisting of STM2457-loaded hydrogel. Subsequently, GOSM-gel and GOS-gel components were administered in a mouse model of CRC, and the fluorescence signal emitted by Cy5.5-labeled STM2457 was observed and recorded using an in vivo fluorescence imaging system. As demonstrated in Fig. 5a and b, the rate of signal degradation in GOSM-gel was slower compared to GOS-gel. About 38 % of the signal emitted by GOSM-gel retained visibility, whereas the signal emitted by GOS-gel was virtually undetectable. Subsequently, the HPLC results revealed a statistically significant increase in serum levels of STM2457 in the GOSM-gel-treated group compared to the IV injection-treated group, as depicted in Fig. 5c. Furthermore, to evaluate in vitro release characteristics, we first prepared FITC labeled STM2457 and verified its FITC labeling through fluorescence imaging, as shown in Fig. S8, the concentrations of STM2457 were evaluated in both the tumor resection bed and serum of mice after the administration of GOSM-gel or intravenous injection of STM2457. In the IF staining analysis conducted on frozen sections of the resected tumor bed margins, the STM2457 fluorescence intensity of day7 and day 14 were statistically significant differences between the GOSM-gel group and IV group indicated that the use of GOSM-gel led to a more sustained release of STM2457 compared to IV injection of STM2457 (Fig. 5d and e). Similarly, under pH 6.5 and pH 7.4, it was demonstrated that STM2457 encapsulated in the GOSM-gel exhibited gradual release over a 21-day period (Fig. 5f). Moreover, the release of STM2457 from the GOSM-gel was faster in pH 6.5 PBS compared to pH 7.4 PBS. Over 21 days, the total amount of STM2457 released was 29.7 % at a pH of 7.4 and 93.4 % at a pH of 6.5. Collectively, the above findings provide compelling evidence of the efficacy of GOSM-gel in preserving the pH-dependent release of STM2457. The pH responsiveness of our hydrogel platform is influenced by a minimum of two mechanisms. The chelating role of calcium within the structure of MBGNs enables the dissolution of Ca from MBGNs under acidic conditions, promoting their disintegration. Additionally, acidic conditions may disrupt the Schiff's base connection, precipitating the hydrogel's disintegration. The degradation of MBGNs occurs when exposed to acidic stimuli, causing them to break down and consequently leading to the release of STM2457 [32]. These findings collectively suggest that the utilization of GOSM-gel enables the extended-release of STM2457 in response to variations in pH, indicating its potential as an effective therapy specifically targeted towards tumors following the surgical removal of colorectal cancer tumors.

**Fig. 5:**
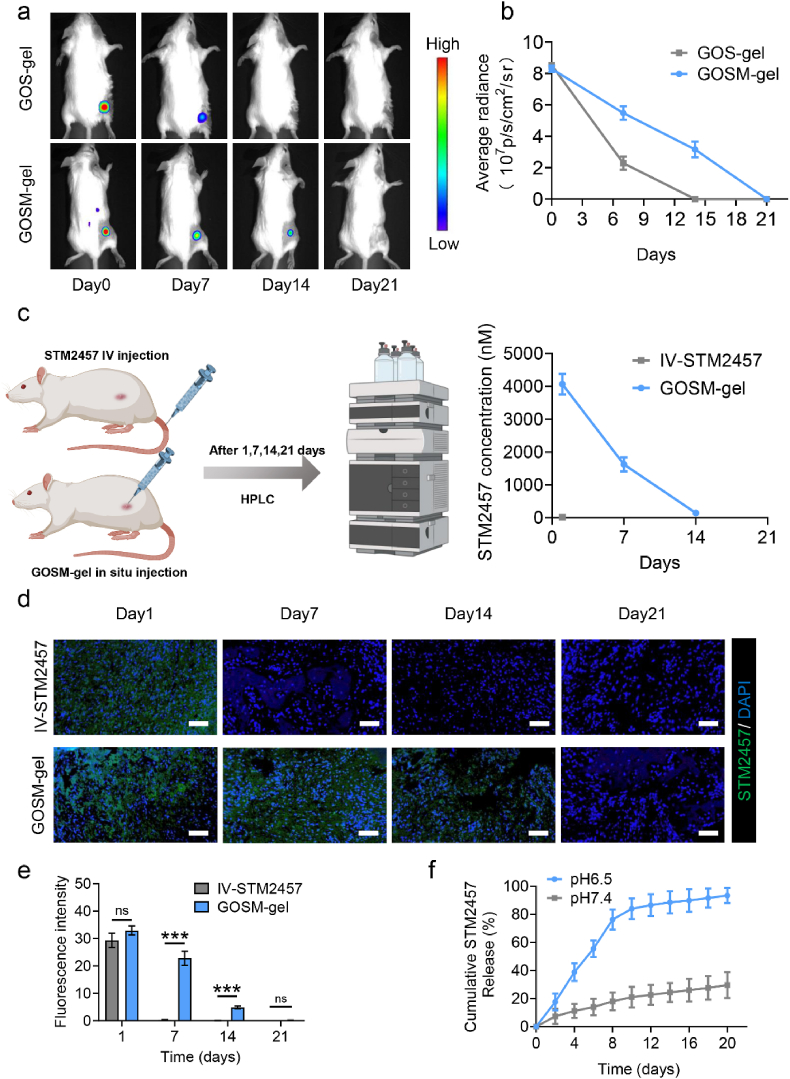
Release of STM2457 by GOSM-gel. (a) The in vivo retention of Cy5.5-labeled STM2457 in GOSM-gel and GOS-gel injected into the tumor resection bed edge was seen using fluorescent IVIS imaging. (b) The quantitative analysis of the in vivo retention profile of Cy5.5-labeled STM2457 (*n* = 3). (c) Serum concentrations of STM2457 following treatment with GOSM-gel or intravenous administration of STM2457 at the indicated time points (*n* = 3). (d) The in vivo retention of FITC-labeled STM2457 in GOSM-gel and GOS-gel injected into the tumor resection site was seen using IF (scale bar = 100 μm). (e) Quantiﬁcation of IF staining of STM2457. (f) Accumulative release of FITC-labeled STM2457 from GOSM-gel at pH 6.5 and pH 7.4 PBS. Data are represented as mean ± SD, with significance levels indicated as follows: ∗*p* < 0.05, ∗∗*p* < 0.01 and ∗∗∗*p* < 0.001.

### 3.5 GOSM-gel reduces m6A modification level to promote in vitro chemotaxis of CAR-NK cells by increasing expression of CXCL9 and CXCL10

The predominant epitranscriptomic modification of mammalian mRNAs, known as m6A, plays a crucial role in controlling the stability, splicing, and translation of mRNAs into proteins in CRC [51]. To investigate the regulatory impact of GOSM-gel on m6A RNA methylation, we measured the levels of m6A RNA in CRC cells using the m6A RNA methylation quantification kit and the m6A dot blot assay after the corresponding treatments. As illustrated in Fig. 6a–c, GOSM-gel significantly reduced the abundance of m6A in CRC cells compared to both the control group and the GOSM-gel group. Simultaneously, we assessed the expression of METTL3 in CRC cells through qPCR and WB after various treatments, revealing a significant downregulation of METTL3 in the GOSM-gel group (Fig. 6d–f). The most substantial reduction in overall m6A mRNA methylation was achieved when STM2457 was loaded to regulate the expression of METTL3. Intriguingly, GOSM-gel exhibited an upregulatory effect on CXCL9 and CXCL10 mRNAs in CRC cells (Fig. 6g–j). CXCL9 and CXCL10 are chemokines known to promote NK cell migration [16], suggesting that GOSM-gel may play a significant role in NK cell recruitment. To validate this hypothesis, we conducted a chemotaxis study using microscopy (Fig. 6k). Our findings indicated that after 8 h of coculture with GOSM-gel, 25.5 % of CAR-NK-92 cells migrated from the upper chamber of the transwell to the bottom chamber, whereas in the presence of the control supernatant, fewer than 10.2 % of CAR-NK-92 cells migrated to the bottom chamber (Fig. 6l). Further supported by colony-formation assay and cell proliferation assay results, the addition of GOSM-gel demonstrated an increase in the cytotoxic activity of CAR-NK cells (Fig. 6m–o). In summary, in vitro experiments confirmed that GOSM-gel, by decreasing the m6A level of CRC cells, upregulates the expression of CXCL9 and CXCL10, thereby enhancing CAR-NK cell migration and cytotoxicity.

**Fig. 6:**
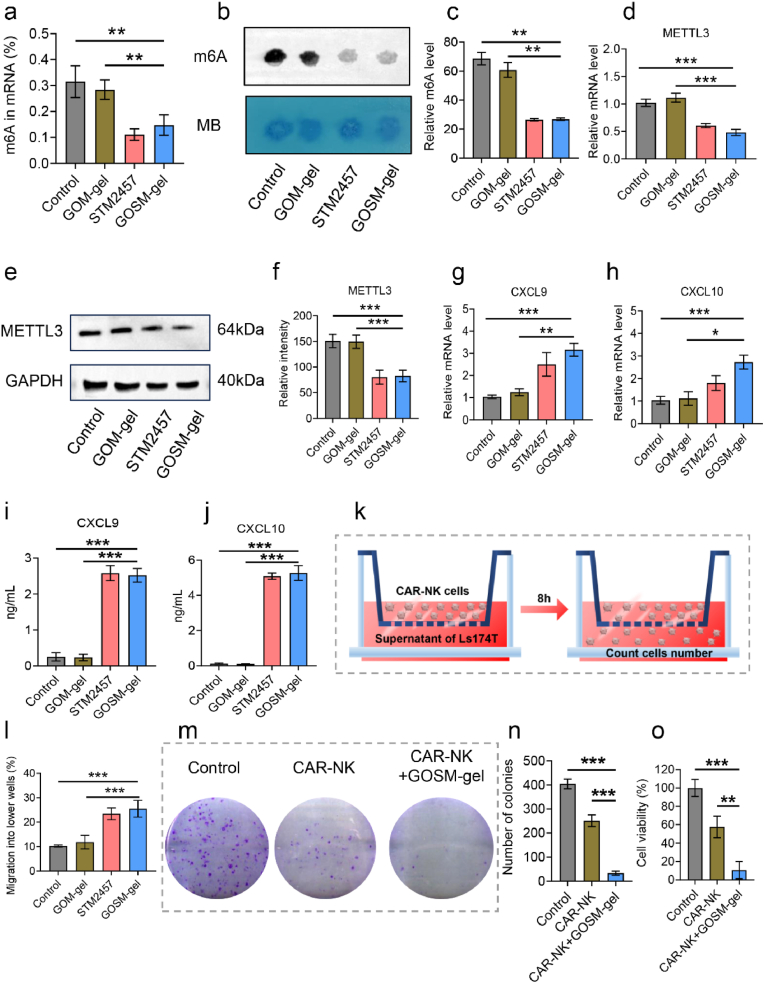
In vitro chemotactic effect of GOSM-gel. (a) Using a m6A RNA methylation quantification reagent, relative m6A mRNA levels were determined for each treatment (*n* = 3). (b) The mRNAs isolated from each treatment were analyzed by dot blot using an anti-m6A antibody, and MB staining served as loading control. (c) The relative m6A content of mRNA was determined for each treatment (*n* = 3). (d) qPCR was used to assess the levels of METTL3 expression in each treatment (*n* = 3). (e) METTL3 protein levels were measured in tumors by WB. (f) Quantiﬁcation of WB of METTL3 (*n* = 3). (g, h) Increased expressions of the CXCL9 and CXCL10 in CRC by GOSM-gel determined by qPCR (*n* = 3). (I, j) Increased expressions of the CXCL9 and CXCL10 in CRC by GOSM-gel by ELISA kits (*n* = 3). (k) A schematic representation of the NK-92 cells transwell migration experiment. (l) NK-92 cell migration into the lower chamber of the transwell in response to various treatment-conditioned media (*n* = 3). (m) Colony-forming assays in Ls174T cells after different treatments. (n) Graphs showing the colonies numbers (*n* = 3). (o) Quantitative results of cell proliferation assay (*n* = 3). Data are represented as mean ± SD, with significance levels indicated as follows: ∗*p* < 0.05, ∗∗*p* < 0.01 and ∗∗∗*p* < 0.001.

### 3.6 The adhesive and hemostatic properties of the GOSM-gel hydrogel

Hydrogel is composed of aldehyde functional groups that can undergo covalent reactions with amino groups present on the surfaces of fresh tissues. This leads to the formation of strong bonds at the interface, enhancing the hydrogel's adherence to tissues. The moist tissue adhesive potential of GOSM-gel, with its aldehyde groups, was thoroughly investigated. The initial step involved evaluating the strength of interfacial tissue attachment through lap-shear adhesion studies. This assessment was then compared to the adhesive properties of GOSM-gel and fibrin glue, commonly used in surgical procedures. As shown in Fig. 7a–c, the GOSM-gel's adhesive strength (103.2 ± 5.93 kPa) was significantly higher than that of fibrin glue (8.8 ± 3.11 kPa) and hydrogel adhesives from prior studies [33]. In order to confirm these findings, burst pressure experiments were conducted. The hydrogel was applied to pig epidermis, and PBS was introduced to detach the hydrogel from the tissue. As shown in Fig. 7d and e the GOSM-gel exhibited a significantly higher average burst pressure compared to fibrin adhesive. Taken together, these findings suggest that GOSM-gel could be a promising sealant. As shown in Fig. 7f, in an ex vivo porcine intestinal model, the GOSM gel demonstrated rapid and effective sealing of a 1.5 cm lesion within 5 min of gelation. The adhesive properties were stable in the liver adhesion model, even after washing for 60 s under running water (Fig. 7g and h). Overall, our study findings indicate that GOSM-gel has a notable capacity to adhere to moist tissues. A growing body of research suggests that intraoperative bleeding following tumor excision can lead to the dissemination of cancer cells into the bloodstream, thereby increasing the likelihood of local tumor recurrence. The use of an effective and prompt hemostasis method during surgical procedures can yield positive outcomes by reducing the risk of tumor recurrence. Manual compression and heat hemostasis are commonly employed in clinical practice as standard techniques for managing intraoperative bleeding. However, these approaches have limited efficacy in anatomically challenging areas. Injectable polymer hydrogels containing anticancer drugs show significant potential in addressing intraoperative bleeding. Consequently, a series of studies were conducted to validate the hemostatic efficacy of the GOSM-gel. In this research, a rat model of partial hepatectomy was utilized to investigate hemostasis, as illustrated in Fig. 7i. Quantitative analysis revealed that the GOSM-gel group exhibited significantly reduced blood loss compared to the other two groups (Fig. 7j and k). To assess the hemostatic properties of GOSM-gel, a model of rat tail amputation hemorrhage was employed, in addition to the rat partial hepatectomy hemorrhage model (Fig. 7l). In this model, the GOSM-gel group demonstrated superior hemostatic properties compared to both the control group and the group treated with commercial gelatin sponges (Fig. 7m and n). Overall, our study findings substantiate that GOSM-gel has the potential to serve as both an anticancer drug delivery platform and an intraoperative hemostatic agent.

**Fig. 7:**
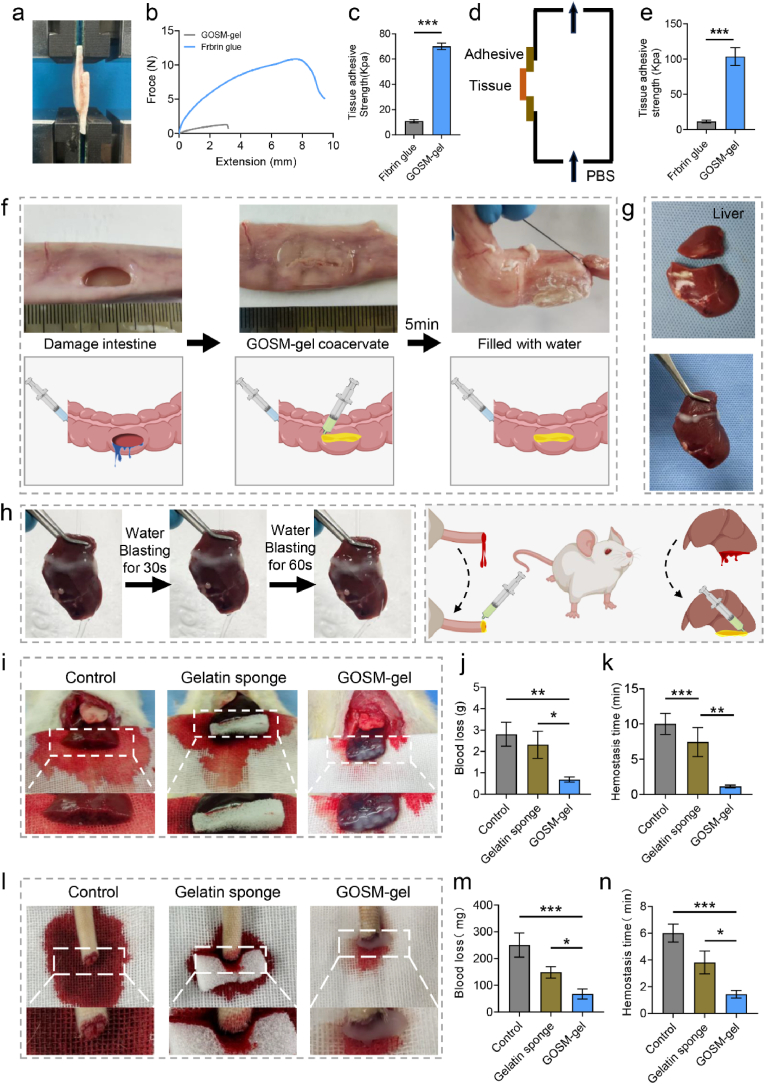
The adhesive and hemostatic properties of GOSM-gel. (a) Schematic diagrams of lap-shear adhesive strength test. (b) Lap-shear adhesion curves of GOSM-gel and fibrin glue applied to porcine epidermis. (c) Comparing the average adhesive strength of GOSM-gel and fibrin glue (*n* = 3). (d) Diagram of the instrument for measuring explosion pressure. (e) Comparing the average release pressure of GOSM-gel and fibrin adhesive (*n* = 3). (f) The GOSM-gel can effectively seal a damaged porcine intestine in vitro. (g) Adhesion of GOSM-gel to liver. (h) Adhesion of GOSM-gel to rat liver under running water for 60 s. (i) Representative photos of rat partial hepatectomy wound bleeding models, quantitative statistics of (j) blood loss and (k) hemostasis time for different groups (*n* = 3). (l) Representative photos of mouse tail amputation bleeding models, quantitative statistics of (m) blood loss and (n) hemostasis time for different groups (*n* = 3). Data are represented as mean ± SD, with significance levels indicated as follows: ∗*p* < 0.05, ∗∗*p* < 0.01 and ∗∗∗*p* < 0.001.

### 3.7 GOSM-gel increases recruitment of NK cells to tumors in an in situ C57BL/6 mouse CRC model

To provide a more practical and translatable assessment of the treatment's efficacy, we had used an in situ tumor model in immunocompetent mice which adequately reflect the therapeutic potential in a realistic and clinically relevant context. To evaluate the therapeutic efficacy in a manner that is more clinically relevant, we employed an in situ tumor model utilizing immunocompetent mice, which more accurately reflects the treatment's potential in a realistic setting. Specifically, MC38-Luc cells were inoculated into the serosal layer of the cecum in C57BL/6 mice. After 7 days of post-inoculation, mice bearing tumors were stratified into four distinct treatment groups: a control group injecting PBS, and three experimental groups injecting GOM-gel, STM2457, and GOSM-gel, respectively. These treatments were directly administered into the established tumors as shown in Fig. 8a. Non-invasive in vivo bioluminescence imaging was utilized to monitor tumor growth across all treatment groups (Fig. 8b). Analysis of the imaging data revealed that the GOSM-gel group demonstrated a significant suppression in tumor growth compared to the other groups that received PBS, GOM-gel and STM2457 (Fig. 8c). On day 20, tumors were harvested from each treatment group for subsequent flow cytometry analysis. Notably, the GOSM-gel group displayed the highest infiltration of NK cells within the tumor microenvironment (Fig. 8d and e). These findings suggest that GOSM-gel mediates its antitumor effects by enhancing NK cells recruitment to the tumor site in an immunocompetent mouse model of in situ colorectal cancer. This immune cell infiltration was contributed to the observed tumor growth inhibition in the GOSM-gel treated group.

**Fig. 8:**
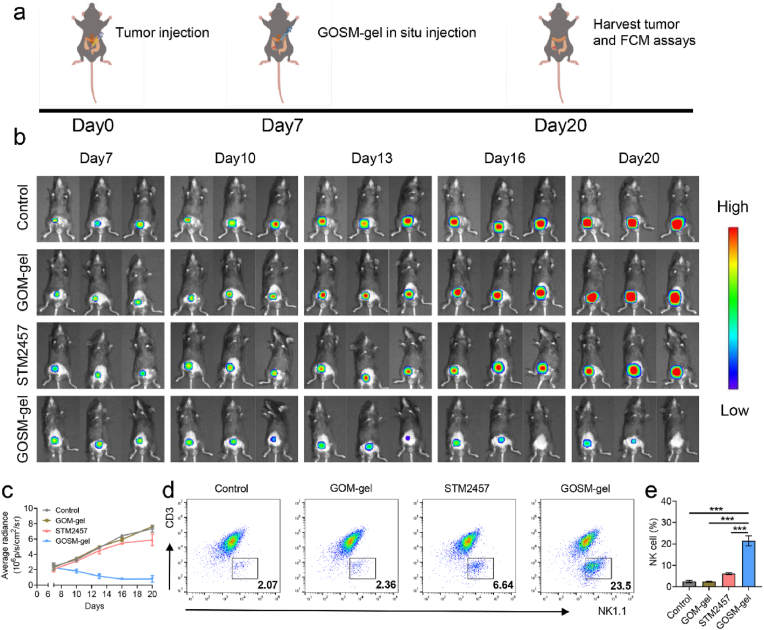
The efficacy of GOSM-gel on in situ colorectal cancer in C57 mice. a) Schematic illustration of the in vivo treatments of the in situ colorectal cancer in C57 mice; b In vivo bioluminescence imaging of the mice bearing MC38-Luc colorectal cancer after different treatments; c) Average tumor growth kinetics in different groups (*n* = 6 for each group); d) Representative flow cytometric analysis images of NK1.1^+^ cells; e) The relative quantification of NK1.1^+^ cells (*n* = 3); Data are represented as mean ± SD, with significance levels indicated as follows: ∗*p* < 0.05, ∗∗*p* < 0.01 and ∗∗∗*p* < 0.001.

### 3.8 Preventing the recurrence of CRC with the GOSM-gel hydrogel

Postresection relapse in CRC is mainly attributed to the existence of CRC cells in the remaining foci [4]. According to previous studies [52,53], an incomplete tumor resection model was established by removing most tumor when the volume of subcutaneous Ls174T tumor reaches 200–300 mm^3^ to evaluate the post-operative therapeutic effect of GOSM-gel. Therefore, additional research was conducted to investigate whether the local administration of GOSM may enhance the efficacy of CAR-NK cell infusion in preventing CRC recurrence after surgery. In Fig. 9a, CRC models in mice were established by subcutaneously injecting 5 × 10^6^ Ls174T-Luc cells per NOD-SCID mice. Following a surgical procedure at day 7 to remove the tumor, the mice were randomly assigned to five groups: the untreated group (no additional treatments), CAR-NK (CAR-NK cell injection alone), CAR-NK-GOM (CAR-NK cell injection with drug-unloaded hydrogel injection onto the tumor resection bed), CAR-NK-IV-STM2457 (CAR-NK cell injection with IV STM2457), and CAR-NK-GOSM (CAR-NK cell injection with GOSM injection). In vivo bioluminescence imaging was employed to track tumor recurrence in each group (Fig. 9b). Based on continuous observation of the photograph (Fig. 9b) taken by the in vivo bioluminescence imaging system at multiple time periods, the residual tumor after resection is essentially consistent with the center position of the fluorescent signal of the recurrent tumor. We can infer that incomplete resection of the positive surgical margin is the cause of tumor recurrence. As shown in Fig. 9c, the untreated group of mice exhibited a relapse rate of 100 % during the 40-day observation period. The relapse rate slightly decreased in the CAR-NK, CAR-NK-GOM, and CAR-NK-IV-STM2457 groups, reaching around 70 %, and significantly dropped to 30 % in the CAR-NK-GOSM group. HE and IHC staining revealed that treatment with GOSM-gel elevated levels of Caspase-3 protein in the CRC model, resulting in a more potent suppressive effect on tumor growth (Fig. 9d and e). Furthermore, among all therapeutic modalities, CAR-NK infusion combined with GOSM-gel injection significantly enhanced the overall survival of mice, as determined by Kaplan-Meier survival analysis with the log-rank test (Fig. 9f). Additionally, weight remained constant in mice from the GOSM-gel group (Fig. 9g), indicating that no additional systemic toxicity was induced by GOSM-gel treatment. Consequently, in situ injection of GOSM-gel significantly improved the curative effect of CAR-NK cell infusion as a therapeutic approach for treating the recurrence of colorectal cancer after surgical removal.

**Fig. 9:**
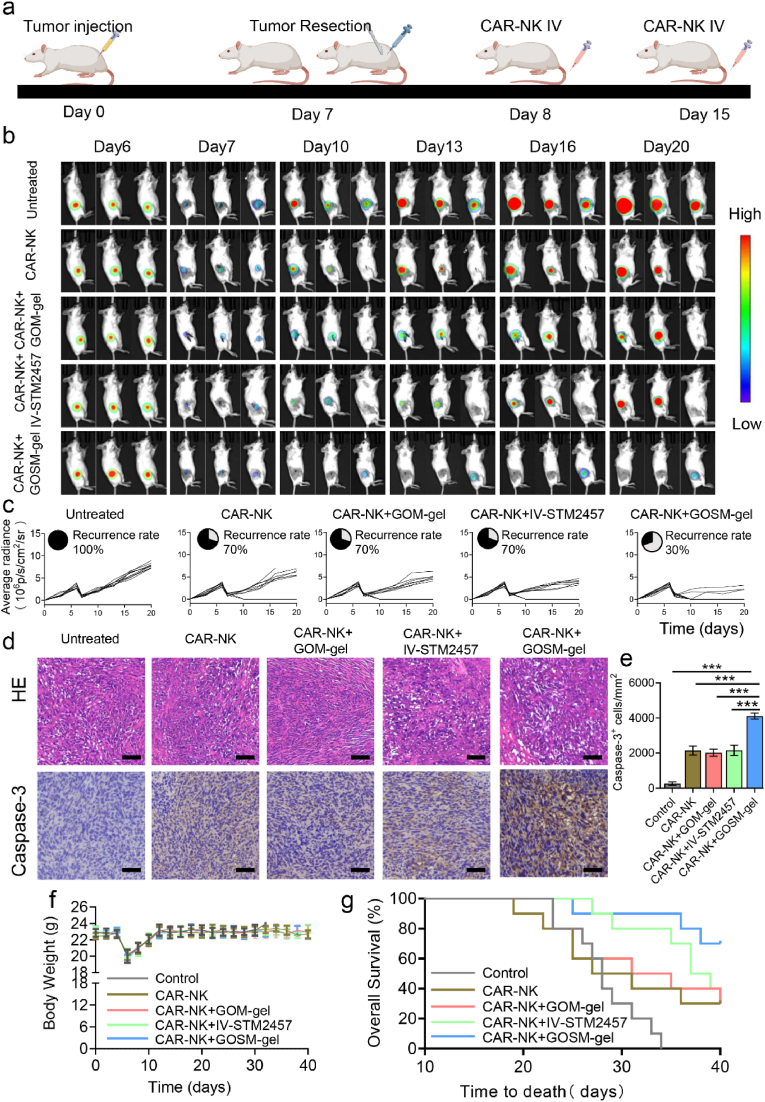
Prevention of CRC recurrence by GOSM-gel. (a) Illustration of the adoptive CAR-NK cell injection and GOSM-gel in a tumor resection CRC model. (b) In vivo bioluminescence imaging showcasing the recurrent tumors after surgical resection of tumor. (c) Kinetics of individual tumor regrowth across distinct treatment groups (*n* = 10). (d) Histochemical and H&E staining of tumor tissues from the various treatment groups (scale bars: 50 μm). (e) Caspase-3 immunohistochemistry quantification (*n* = 3). (f) Weight variations of mice subjected to different treatment modalities (*n* = 10). (g) Weight variations of mice subjected to different treatment modalities. (*n* = 10). Data are represented as mean ± SD, with significance levels indicated as follows: ∗*p* < 0.05, ∗∗*p* < 0.01 and ∗∗∗*p* < 0.001.

### 3.9 GOSM-gel decreases the level of m6A modification to improve the in vivo therapeutic effects of CAR-NK cells on CRC by stimulating tumor infiltration

To demonstrate that GOSM-gel can enhance the infiltration of CAR-NK cells into tumors, 15 mice models of CRC were randomly assigned to the previously described five groups. On day 15, recurrent tumors were surgically removed from mice in each experimental group and analyzed using IF staining and IHC staining techniques. As presented in Fig. 10a–c, GOSM-gel treatment upregulated CD56 protein levels in the CRC model, resulting in an increased effect on adoptively transferred CAR-NK cells' tumor infiltration. The flow cytometry analysis consistent with histological findings, it was found that the proportion of NK cells was higher in the GOSM-gel group (Fig. S9). To explore the mechanism, we measured the amounts of m6A RNA in the CRC cells using the m6A dot blot assay and the m6A RNA methylation quantification kit after various treatments to investigate if GOSM-gel regulates m6A RNA methylation. As shown in Fig. 10d–f, GOSM-gel significantly reduced the abundance of m6A in CRC cells compared to the other groups. Simultaneously, we measured the expression of METTL3 in the tumor using qPCR and WB after various treatments, and the GOSM-gel group significantly downregulated the expression of METTL3 (Fig. 10g–i). Consistently, GOSM-gel upregulated m6A-modified CXCL9 and CXCL10 in serum (Fig. 10j–m). It can be inferred that GOSM-gel could enhance CAR-NK cell infiltration into tumors by increasing the concentrations of CXCL9 and CXCL10 in the serum. In conclusion, the findings of this study validate the ability of our hydrogel platform to enhance NK cell infiltration into tumors during adoptive NK cell therapy.

**Fig. 10:**
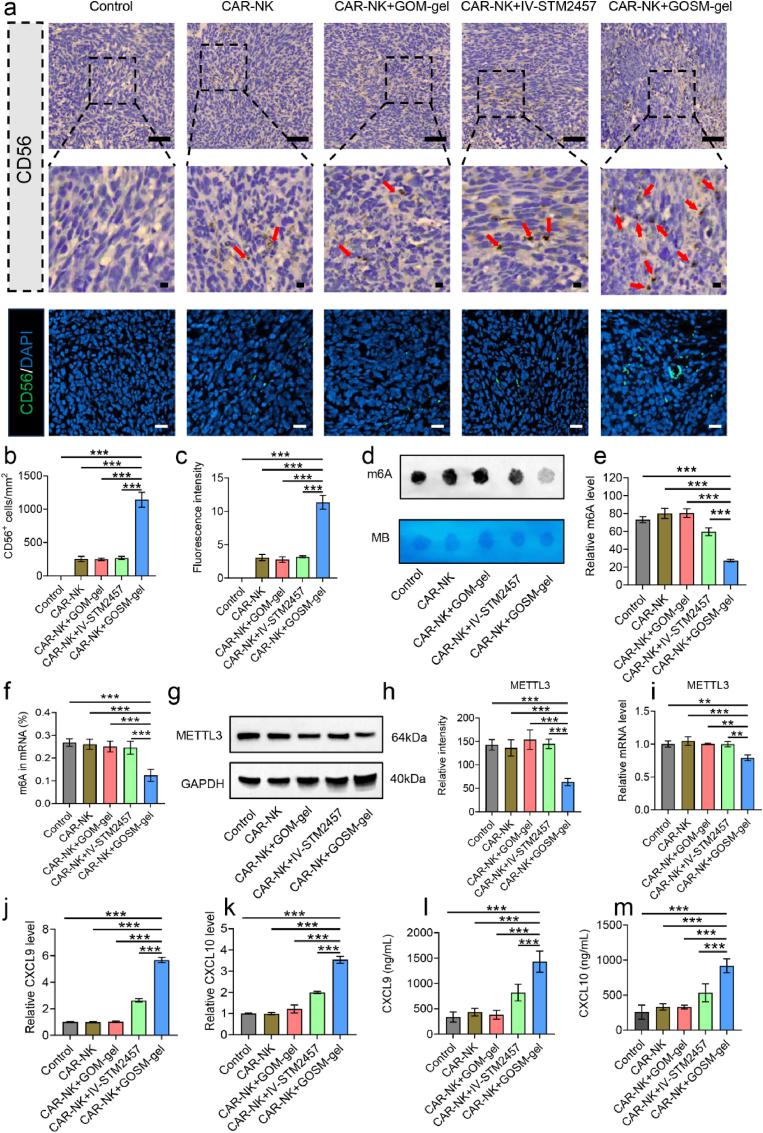
Chemotactic effect of GOSM-gel in vivo. (a) The number of CAR-NK cells that have infiltrated tumor sections was quantified using immunohistochemical and IF staining of CD56. (b) Quantiﬁcation of immunohistochemistry of CD56 (*n* = 3). (c) Quantiﬁcation of IF staining of CD56 (*n* = 3). (d)The mRNAs isolated from each treatment were analyzed by dot blot using an anti-m6A antibody, and MB staining served as a loading control. (e) The relative m6A content of mRNA was determined for each treatment (*n* = 3). (f) Using a m6A RNA methylation quantification reagent, relative m6A mRNA levels were determined for each treatment (*n* = 3). (g) Tumor METTL3 protein levels were determined by WB. (h) Quantiﬁcation of WB of METTL3 (*n* = 3). (i) qPCR was used to assess the levels of METTL3 expression in each treatment (*n* = 3). (j, k) Using qPCR to determine the levels of CXCL9 and CXCL10 mRNA expression in tumor (*n* = 3). (l, m) using an ELISA kits to determine expressions of the CXCL9 and CXCL10 in tumor (*n* = 3). Data are represented as mean ± SD, with significance levels indicated as follows: ∗*p* < 0.05, ∗∗*p* < 0.01 and ∗∗∗*p* < 0.001.

## 4 Conclusion

In summary, to prevent CRC recurrence following resection, we designed an injectable hydrogel incorporating a METTL3 inhibitor. It exhibits key characteristics, including injectability, biocompatibility, and sustained drug release. In vitro, the gel can suppress n6-methyladenosine RNA methylation in CRC, it leads to an increase in CXCL9 and CXCL10 expression, promoting CAR-NK cells migration to eliminate CRC cells. Meanwhile, the gel can be readily injected into the resection margin of tumor, with its intermolecular hydrogen bonding and dynamic covalent Schiff base network, rapidly forming an adhesive and hemostasis gel that can adapt to complex clinical scenarios. The gel significantly enhances the therapeutic efficacy of CAR-NK cell infusion to prevent CRC recurrence following surgery in a CRC mouse model. This study offers valuable insights into the potential applications of CAR-NK cell adoptive therapy and demonstrates the feasibility of utilizing a hydrogel-based delivery system to effectively reduce the recurrence of colorectal cancer after surgical removal.

## CRediT authorship contribution statement

**Zilin Tan:** Writing – original draft, Validation, Methodology, Investigation, Formal analysis, Conceptualization. **Liangjie Tian:** Validation, Methodology, Formal analysis. **Yang Luo:** Validation, Methodology, Formal analysis. **Kexin Ai:** Validation, Methodology, Formal analysis. **Xuehua Zhang:** Validation, Methodology, Formal analysis. **Haitao Yuan:** Visualization, Investigation. **Jinfan Zhou:** Methodology. **Guangyao Ye:** Validation, Formal analysis. **Shuofei Yang:** Visualization, Resources. **Ming Zhong:** Visualization, Resources, Data curation. **Gaohua Li:** Supervision, Methodology, Funding acquisition. **Yanan Wang:** Writing – review & editing, Supervision, Project administration, Methodology, Funding acquisition, Data curation, Conceptualization.

## Ethics approval and consent to participate

All animal experiments were carried out in the Animal Experimental Center of Nanfang Hospital. All operations were trained, and the experimental protocol was reviewed and approved by the Ethics Committee of Southern Medical University.

## Declaration of competing interest

The authors declare no conflict of interest.

## Figures and Tables

**figs1:**
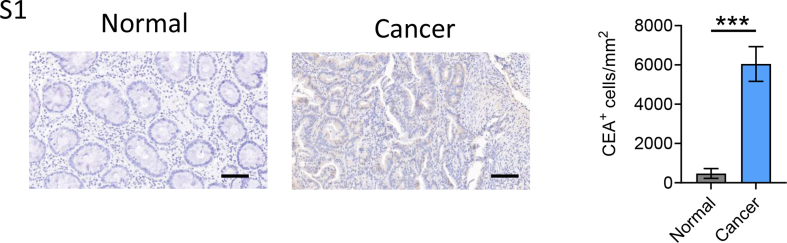


**figs2:**
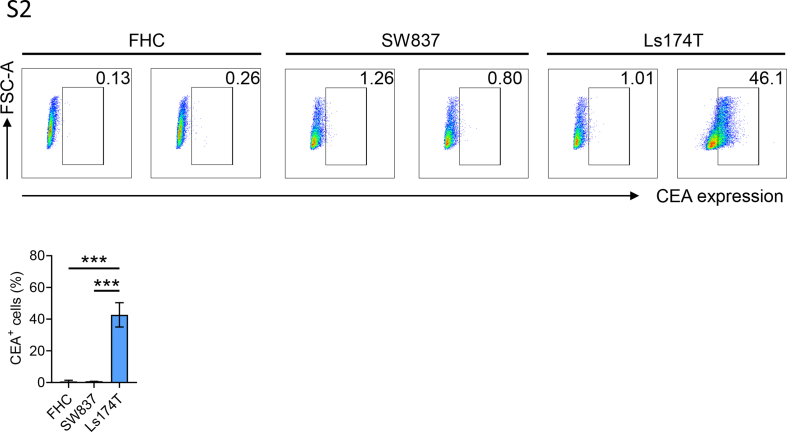


**figs3:**
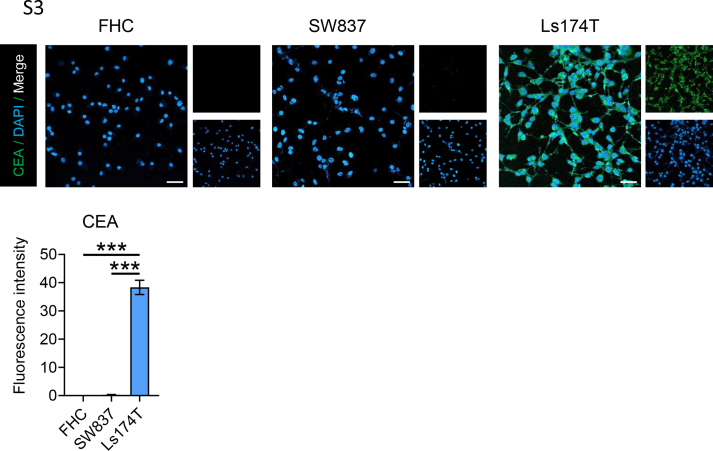


**figs4:**
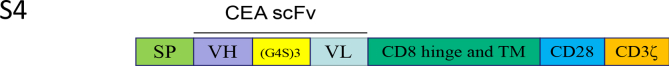


**figs5:**
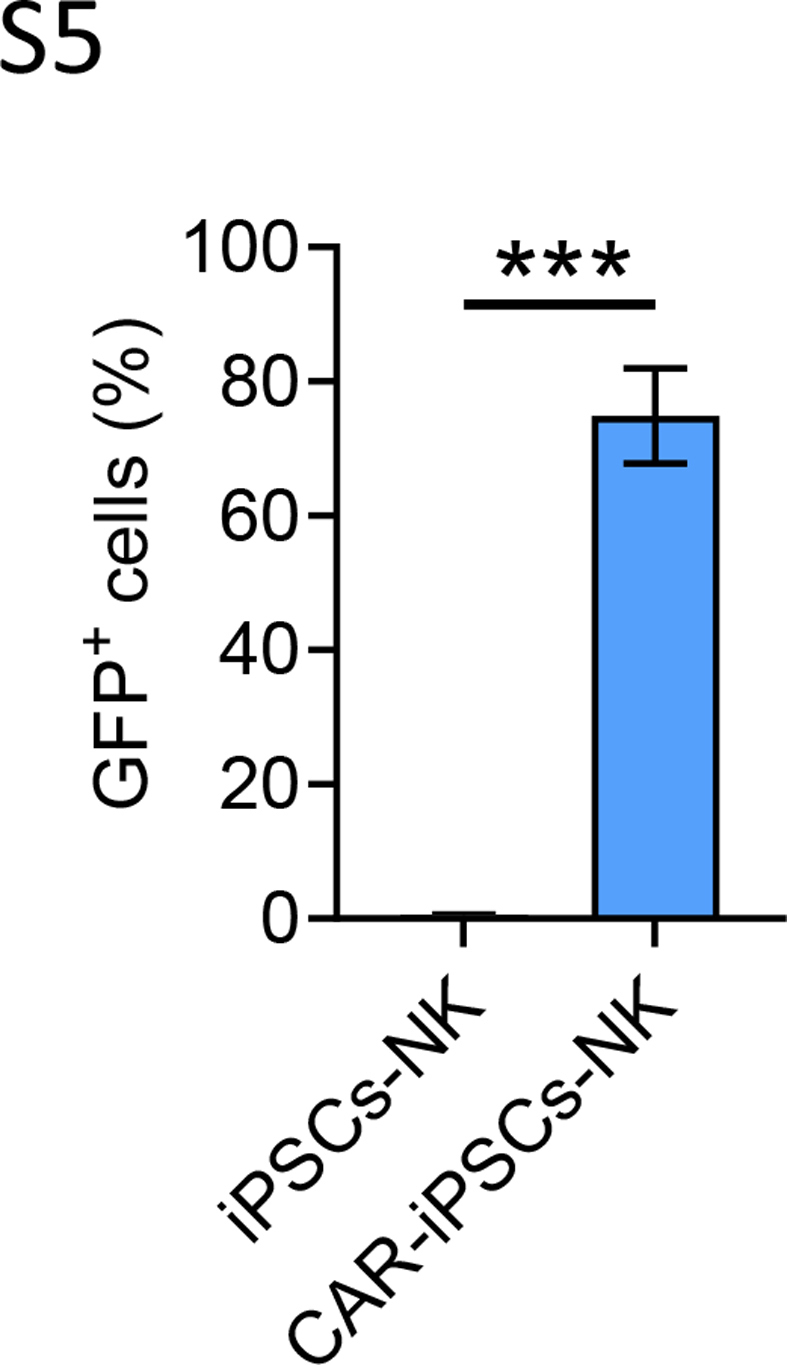


**figs6:**
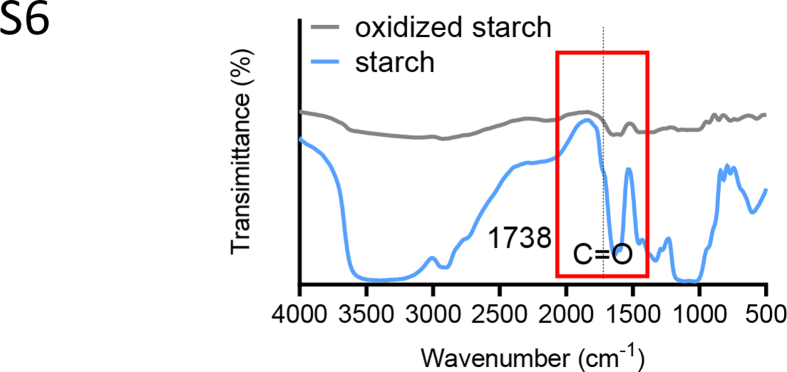


**figs7:**
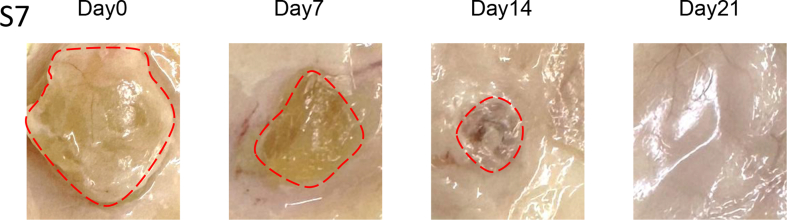


**figs8:**
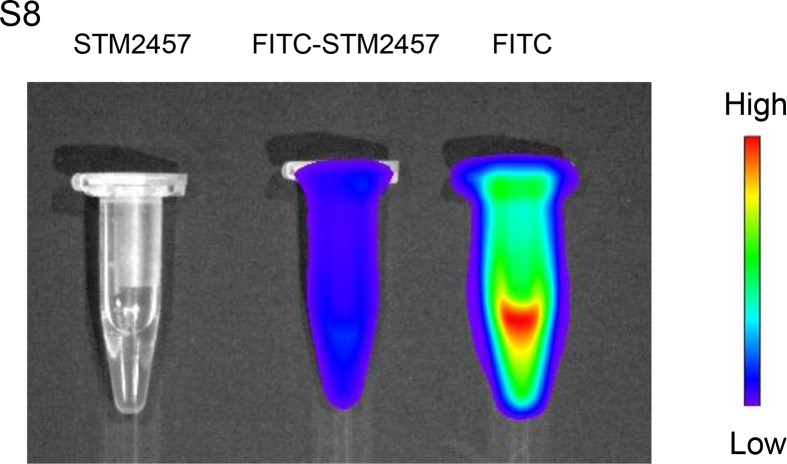


**figs9:**
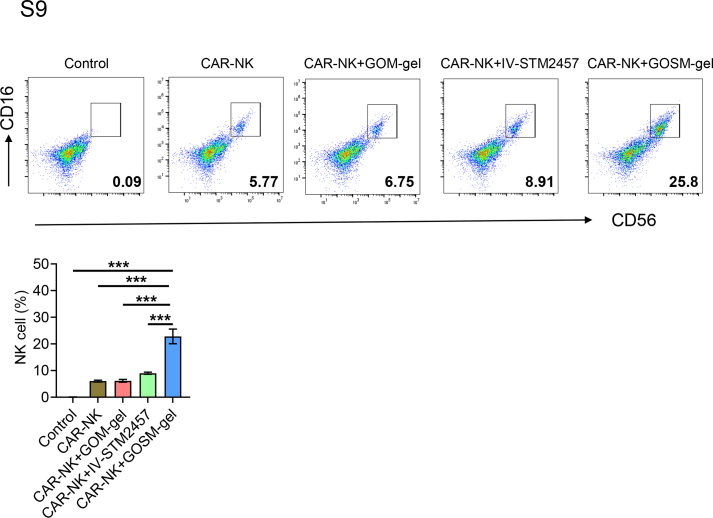


**figs10:**
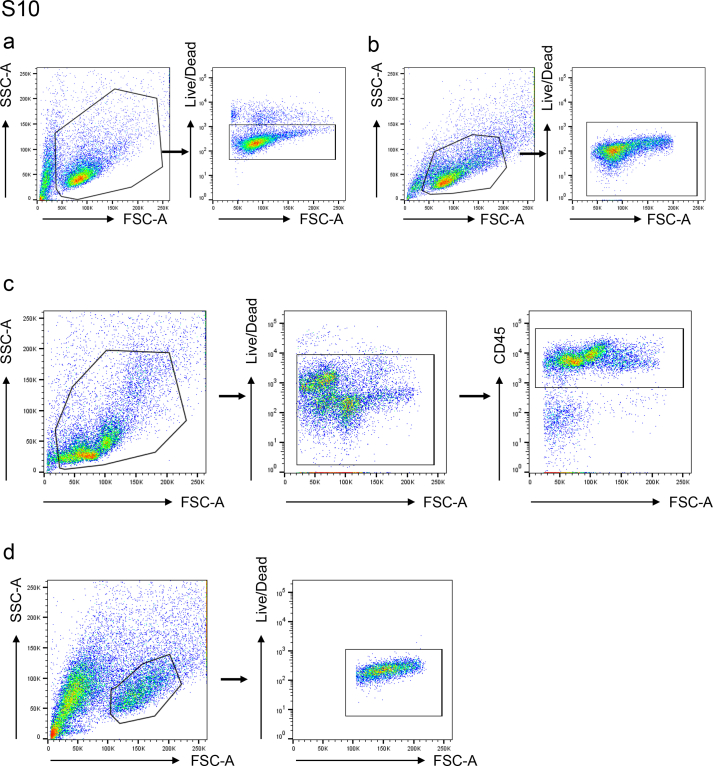


**figs11:**
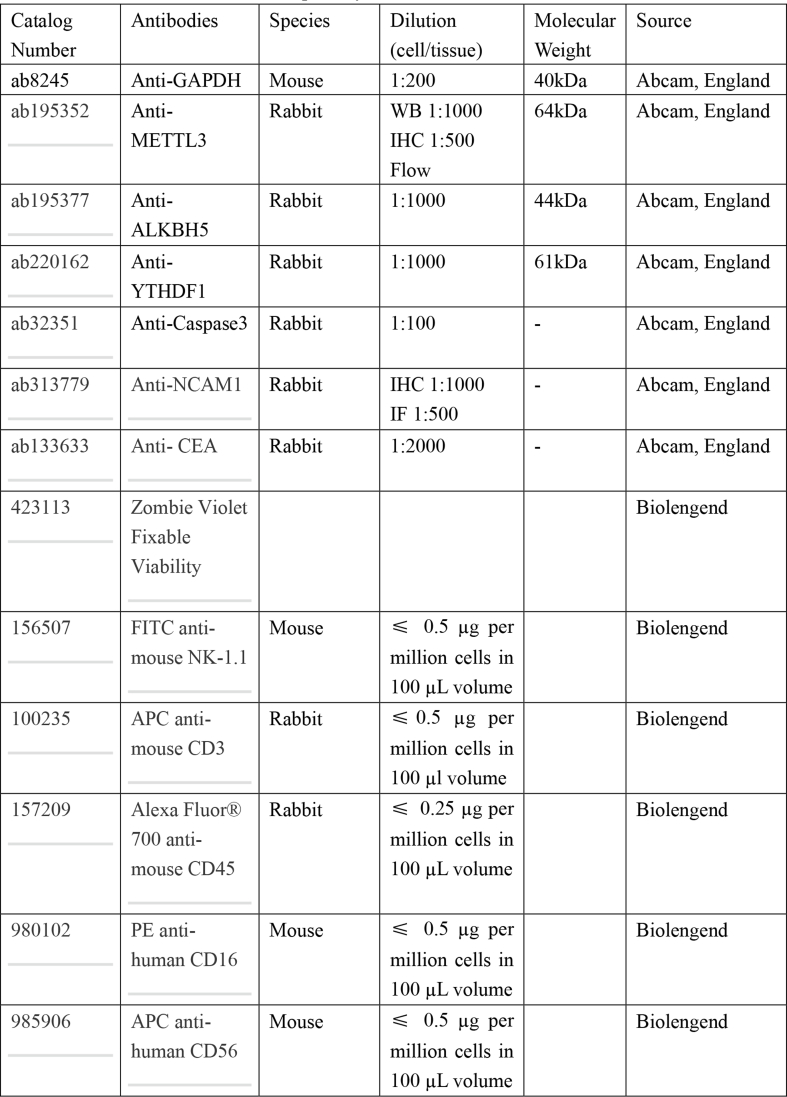


**figs12:**